# Mining patterns of comorbidity evolution in patients with multiple chronic conditions using unsupervised multi-level temporal Bayesian network

**DOI:** 10.1371/journal.pone.0199768

**Published:** 2018-07-12

**Authors:** Syed Hasib Akhter Faruqui, Adel Alaeddini, Carlos A. Jaramillo, Jennifer S. Potter, Mary Jo Pugh

**Affiliations:** 1 Department of Mechanical Engineering, The University of Texas at San Antonio, San Antonio, TX, United States of America; 2 South Texas Veterans Health Care System, San Antonio, TX, United States of America; 3 Department of Psychiatry, University of Texas Health Science Center at San Antonio, San Antonio, TX, United States of America; 4 VA Salt Lake City Health Care System, Salt Lake City, UT, United States of America; Boston University & VA Boston Healthcare System, UNITED STATES

## Abstract

Over the past few decades, the rise of multiple chronic conditions has become a major concern for clinicians. However, it is still not known precisely how multiple chronic conditions emerge among patients. We propose an unsupervised multi-level temporal Bayesian network to provide a compact representation of the relationship among emergence of multiple chronic conditions and patient level risk factors over time. To improve the efficiency of the learning process, we use an extension of maximum weight spanning tree algorithm and greedy search algorithm to study the structure of the proposed network in three stages, starting with learning the inter-relationship of comorbidities within each year, followed by learning the intra-relationship of comorbidity emergence between consecutive years, and finally learning the hierarchical relationship of comorbidities and patient level risk factors. We also use a longest path algorithm to identify the most likely sequence of comorbidities emerging from and/or leading to specific chronic conditions. Using a de-identified dataset of more than 250,000 patients receiving care from the U.S. Department of Veterans Affairs for a period of five years, we compare the performance of the proposed unsupervised Bayesian network in comparison with those of Bayesian networks developed based on supervised and semi-supervised learning approaches, as well as multivariate probit regression, multinomial logistic regression, and latent regression Markov mixture clustering focusing on traumatic brain injury (TBI), post-traumatic stress disorder (PTSD), depression (Depr), substance abuse (SuAb), and back pain (BaPa). Our findings show that the unsupervised approach has noticeably accurate predictive performance that is comparable to the best performing semi-supervised and the second-best performing supervised approaches. These findings also revealed that the unsupervised approach has improved performance over multivariate probit regression, multinomial logistic regression, and latent regression Markov mixture clustering.

## Introduction

For nearly a decade, clinicians caring for Veterans with traumatic brain injury (TBI) have described multimorbidity among those with persistent post-concussion symptoms and other commonly occurring comorbidities. This constellation was first described as the Polytrauma Clinical Triad (PCT), and included TBI, post-traumatic stress disorder (PTSD) and pain [1]. At the national level, approximately six percent of Post-9/11 Veterans were diagnosed with TBI, PTSD and pain in 2009 [2]. The number of Veterans receiving care for PCT were increased by a total of 144% over the triennium, and the health care cost that was four times higher than similar Post-9/11 Veterans without TBI [3]. PCT were also associated with sleep disturbance [4], suicide [5]. Miller et al. [6] showed that service members with mild TBI are at increased risk for addiction-related disorders including alcohol and nicotine. They also observed that mild TBI is distinguished from moderate to severe TBI in terms of timing of the risk, indicating a need for rigorous TBI clinical screening. Corrigan et al. [7] identified seven clusters of lifetime history of TBI for substance abuse problem. These clusters were characterized by injury severity, age of injury occurrence, and periods of repeated TBI. The clusters differed in their contribution to predict future consequences, which suggests an underlying complexity of the medical history that results in substance abuse. Adams et al. [8] conducted a path based analysis to examine the association of binge alcohol drinking with TBI and PTSD. The study sample included 6,824 military personnel and revealed that PTSD and a prior history of TBI both had a direct effect on binge drinking.

Lippa et al. [9] used factor analysis to identify patterns of comorbidity in a sample of 255 previously deployed Post-9/11 service members and veterans who participated in a structured clinical interview. They found that over 90% of the patients had psychiatric conditions, and approximately half had three or more conditions. They also identified four clinically relevant psychiatric and behavioral factors, including deployment trauma factor, somatic factor, anxiety factor, and substance abuse factor, that account for 76.9% of the variance in the data. They concluded that depression, PTSD, and a history of military mild TBI can comprise a harmful combination associated with high risk for substantial disability. Using a broader range of comorbid conditions, Pugh, et al. [10] used latent class analysis (LCA) to identify longitudinal comorbidity phenotypes in previously deployed Post-9/11 Veterans based on diagnoses received during the first three years of care in the Veterans Health Administration (VA). In analyses stratified by sex, they found five phenotypes that were consistent in men and women: Healthy, Chronic Disease, Pain, Mental Health, and Polytrauma Clinical Triad (Mental Health, TBI and Pain). These subgroups demonstrated increasing likelihood of having relevant diagnoses over time. Pugh et al. [11] showed that these comorbidity phenotypes are associated with measures of community reintegration, with individuals in the PCT and Mental Health groups being significantly more likely to report difficulty in the transition from military to civilian life, lower levels of social support, and higher rates of unemployment. Alaeddini, et al. [12] developed a Latent Regression Markov Mixture Clustering (LRMCL) algorithm to identify major transitions of four MCC that include hypertension (HTN), depression, PTSD, and back pain in a cohort of 601,805 Iraq and Afghanistan war Veterans (IAVs). The LRMCL algorithm was also able to predict the exact status of comorbidities about 48% of the time.

Zador et al. [13] used logistic regression to predict TBI outcome in the dataset of the corticosteroid randomization after significant head injury (CRASH), where they utilized a Bayesian network to assess the dependencies between predictors of the model. This gave a strong insight in formalizing clinical intuition for the demographic predictors being used. Zador et al. [14] employed a similar approach based on Bayesian networks to visualize the probabilistic associations between outcome predictors of acute aneurysmal subarachnoid hemorrhage. Cai et al. [15] also established a Bayesian network using a tree-augmented naïve Bayes algorithm to mine relationships between factors influencing hepatocellular carcinoma after hepatectomy. While Bayesian networks can be used to predict disease prevalence, it is also, feasible to use them for providing accurate personalized survival estimates and treatment selection for patient specific variables. Sesen et al. [16] used such a Bayesian network model to create a decision support system for lung cancer care. Forsberg et al. [17] developed and trained a machine-learned Bayesian belief network model to estimate survival time in patients with operable skeletal metastases using candidate features based on historical data. Stojadinovic et al. [18] also used a machine-learned Bayesian belief network to provide clinical decision support in estimating overall survival among colon cancer patients based on a set of prognostic factors at 12-, 24-, 36-, and 60-month post-treatment follow-up. Moreover, Bayesian Networks can be used to study the evolutionary course of multiple disorders. Lappenschaar et al. [19] used a large dataset to develop a multilevel temporal Bayesian networks to model the progression of six chronic cardiovascular conditions.

While interesting, most of these studies have been cross-sectional or focus on a relatively short period of time. Moreover, while these methods describe general comorbidity phenotypes, they do not provide insight into the impact of TBI and comorbid conditions on specific adverse outcomes. In this paper, we develop an unsupervised Multi-level Temporal Bayesian Network (MTBN) from big data to identify the relationships among emergence of five deployment related conditions (TBI, PTSD, Depr, SuAb, and BaPa), and patient level risk factors (race, gender, age, education and marital status) over time. We also use a Longest Path Algorithm (LPA) to identify the most probable sequence of comorbidities emerging from and/or leading to specific chronic conditions. Moreover, we demonstrate the performance of the proposed unsupervised MTBN in comparison with the semi-supervised and supervised MTBNs, as well three baseline methods in the literature, including multivariate probit regression [20], multinomial logistic regression [21], and Latent Regression Markov Mixture Clustering (LRMCL) [12].

## Methods

### Study population

The proposed study uses de-identified data from a large national cohort of patients (N = 608,503) who were deployed in support of the wars in Afghanistan and Iraq and who entered care in the Department of Veterans Affairs between 2002 and 2011 and who received care at least once a year in three different years between 2002-2015. For the purpose of this analysis, only patients with care each year for the first five consecutive years after entering VA care were included (n = 257,633). Dropout may result from not requiring care, dropping out of VA care, or death.

Individuals in the cohort were identified using the roster of veterans who had been previously deployed in support of Operations Enduring Freedom, Iraqi Freedom, and New Dawn (OEF/OIF/OND roster). The inpatient and outpatient data were then obtained from the VA national databases in Austin Texas. Data included ICD-9-CM diagnosis codes documented during the course of VA care, during each inpatient or outpatient encounter.

This study received institutional review board approval from the University of Texas Health Science Center at San Antonio and the Bedford VA Hospital with a waiver of informed consent.

### Measures

#### Diagnosed health conditions

We used ICD-9-CM codes from inpatient and outpatient data (excluding ancillary and telephone care) to identify Traumatic brain injury (TBI), Post Traumatic Disorder (PTSD), Depression (Depr), substance abuse (SuAb), Back pain (BaPa) using validated published algorithms [22]. PTSD, SA, and BP required two diagnoses at least seven days apart, while TBI, which is an acute injury required only a single diagnosis. Each condition was coded as “0” or “1” for each year of care, with 1 indicating a diagnosis for that condition regardless of the number of instances for which each condition was diagnosed (See S1 Table for ICD-9 Codes for the considered conditions in this manuscript). Table 1 illustrates the prevalence of the five comorbidities in the final dataset based on the first five years of care in the VA.

**Table 1:** The prevalence of the five comorbidities in the final dataset based on the five years of care.

TBI	PTSD	BaPa	SuAB	Depr	Year_1	Year_2	Year_3	Year_4	Year_5
0	0	0	0	0	137503	109626	105294	103233	103950
					53.37%	42.55%	40.87%	40.07%	40.35%
0	0	0	0	1	10920	12382	12051	11866	11486
					4.24%	4.81%	4.68%	4.61%	4.46%
0	0	0	1	0	3738	4038	3923	3800	3594
					1.45%	1.57%	1.52%	1.47%	1.40%
0	0	0	1	1	2015	2378	2435	2397	2376
					0.78%	0.92%	0.95%	0.93%	0.92%
0	0	1	0	0	30082	32336	31350	30309	29622
					11.68%	12.55%	12.17%	11.76%	11.50%
0	0	1	0	1	4481	5898	5944	5987	5895
					1.74%	2.29%	2.31%	2.32%	2.29%
0	0	1	1	0	1076	1321	1257	1291	1231
					0.42%	0.51%	0.49%	0.50%	0.48%
0	0	1	1	1	656	969	1010	1079	1030
					0.25%	0.38%	0.39%	0.42%	0.40%
0	1	0	0	0	16570	20550	22515	23350	23414
					6.43%	7.98%	8.74%	9.06%	9.09%
0	1	0	0	1	10084	13313	14803	15201	15513
					3.91%	5.17%	5.75%	5.90%	6.02%
0	1	0	1	0	3015	4363	4899	5352	5459
					1.17%	1.69%	1.90%	2.08%	2.12%
0	1	0	1	1	3542	5152	5844	6173	6366
					1.37%	2.00%	2.27%	2.40%	2.47%
0	1	1	0	0	7878	11031	11962	12641	12953
					3.06%	4.28%	4.64%	4.91%	5.03%
0	1	1	0	1	5539	8619	9576	10266	10899
					2.15%	3.35%	3.72%	3.98%	4.23%
0	1	1	1	0	1355	2088	2438	2696	2879
					0.53%	0.81%	0.95%	1.05%	1.12%
0	1	1	1	1	1755	3096	3554	4055	4252
					0.68%	1.20%	1.38%	1.57%	1.65%
1	0	0	0	0	2834	2515	2000	1699	1562
					1.10%	0.98%	0.78%	0.66%	0.61%
1	0	0	0	1	514	500	448	403	339
					0.20%	0.19%	0.17%	0.16%	0.13%
1	0	0	1	0	198	178	161	133	121
					0.08%	0.07%	0.06%	0.05%	0.05%
1	0	0	1	1	115	137	122	99	85
					0.04%	0.05%	0.05%	0.04%	0.03%
1	0	1	0	0	1528	1352	922	876	793
					0.59%	0.52%	0.36%	0.34%	0.31%
1	0	1	0	1	396	387	316	293	260
					0.15%	0.15%	0.12%	0.11%	0.10%
1	0	1	1	0	116	108	74	60	81
					0.05%	0.04%	0.03%	0.02%	0.03%
1	0	1	1	1	86	86	76	75	68
					0.03%	0.03%	0.03%	0.03%	0.03%
1	1	0	0	0	2937	3303	3034	2916	2639
					1.14%	1.28%	1.18%	1.13%	1.02%
1	1	0	0	1	1858	2293	2253	2187	1990
					0.72%	0.89%	0.87%	0.85%	0.77%
1	1	0	1	0	565	787	782	801	764
					0.22%	0.31%	0.30%	0.31%	0.30%
1	1	0	1	1	834	1182	1195	1216	1171
					0.32%	0.46%	0.46%	0.47%	0.45%
1	1	1	0	0	2429	2995	2878	2677	2435
					0.94%	1.16%	1.12%	1.04%	0.95%
1	1	1	0	1	1804	2741	2518	2452	2396
					0.70%	1.06%	0.98%	0.95%	0.93%
1	1	1	1	0	535	678	733	702	714
					0.21%	0.26%	0.28%	0.27%	0.28%
1	1	1	1	1	675	1231	1266	1348	1296
					0.26%	0.48%	0.49%	0.52%	0.50%

#### Demographic characteristics

Sociodemographic characteristics included age at VA entry, sex, race/ethnicity and education. Age was identified during the first year of VA care. Based on our prior work, we categorized age as 18-30, 31-40, 41-50, and 51 and older. Sex, race/ethnicity (White, African American, Hispanic, Asian/Pacific Islander, Native American, unknown) and education (less than high school, high school graduate, some college, college graduate, post-college education) at the time of leaving the military were obtained from the OEF/OIF/OND Roster; missing values for race and sex were supplemented from VA data. Marital status was obtained from VA data (Table 2).

**Table 2:** Demographics of the patients included in the study.

Sl No.	Race	Gender		Marital Status		Age Group				Education					
		Male	Female	Married	Un-Married	18-30	31-40	41-50	51- Rest	Unknown	< High School	High School	Some College	College Graduate	Post College
1	White	148355	19183	74487	93051	96799	36003	26167	8569	2334	2037	129921	16743	12024	4479
		57.58%	7.45%	28.91%	36.12%	37.57%	13.97%	10.16%	3.33%	0.91%	0.79%	50.43%	6.50%	4.67%	1.74%
2	Black	35758	11828	23308	24278	20047	12468	12710	2361	658	504	37506	4819	3160	939
		13.88%	4.59%	9.05%	9.42%	7.78%	4.84%	4.93%	0.92%	0.26%	0.20%	14.56%	1.87%	1.23%	0.36%
3	Hispanic	25373	4232	14523	15082	17016	6606	4758	1225	386	360	23592	2933	1893	441
		9.85%	1.64%	5.64%	5.85%	6.60%	2.56%	1.85%	0.48%	0.15%	0.14%	9.16%	1.14%	0.73%	0.17%
4	Asian	5639	981	3067	3553	3235	1361	1564	460	131	60	4732	598	879	220
		2.19%	0.38%	1.19%	1.38%	1.26%	0.53%	0.61%	0.18%	0.05%	0.02%	1.84%	0.23%	0.34%	0.09%
5	Native	3081	707	1747	2041	2115	925	564	184	60	60	3004	376	217	71
		1.20%	0.27%	0.68%	0.79%	0.82%	0.36%	0.22%	0.07%	0.02%	0.02%	1.17%	0.15%	0.08%	0.03%
6	Unknown	2135	361	1346	1150	1062	625	673	136	51	22	1808	287	223	105
		0.83%	0.14%	0.52%	0.45%	0.41%	0.24%	0.26%	0.05%	0.02%	0.01%	0.70%	0.11%	0.09%	0.04%

### Bayesian networks

**Fig 1:**
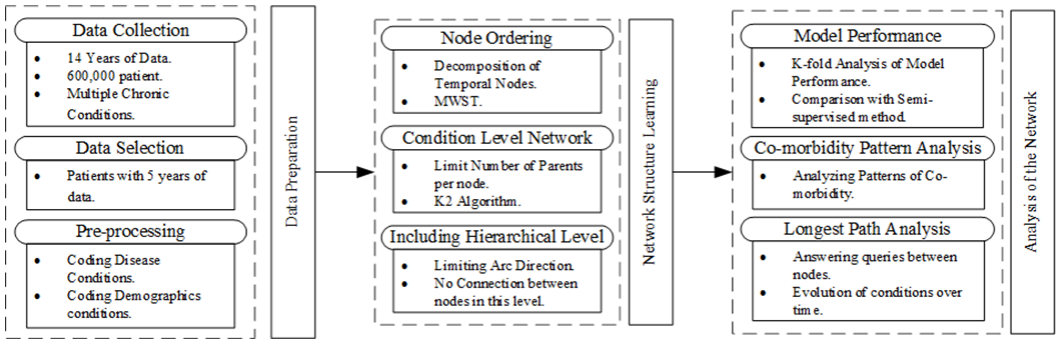
Framework. General Scheme of the proposed method.

In this section, we describe the proposed unsupervised approach along with the supervised and semi-supervised approaches for structure learning in Bayesian networks to mine patterns of comorbidity evolution in patients with Multiple Chronic Conditions (MCC). We also present a longest path algorithm to identify the most likely path to evolution of a chronic condition. This is one of the first studies to demonstrate the inherent association among chronic conditions and demographics for evolution of new conditions. Fig 1 shows the general scheme of the proposed approach.

Bayesian network [23–27] is a probabilistic graphical model that represents a set of variables and their conditional dependencies via a directed acyclic graph (DAG). In this study two sets of low-level and high-level variables are considered: (1) low level binary variables representing having or not having a chronic condition, namely TBI, PTSD, BaPa, Depr, and SuAb, and (2) high-level discrete variables representing demographic factors, namely, race, gender, marital status, age group and education. When the structure of a Bayesian network (DAG) in known, the joint probability distribution over the random variables can be derived according to the dependencies represented in the graph:

$$\begin{array}{c} P\left(X_{1},.....,X_{n}\right)=\prod_{i=1}^{n}P\left(X=x_{k}^{t}|Pa\left(X=x_{k}^{t}\right)\right) \end{array}$$ (1)

Eq (1) can be used to calculate the likelihood and estimate the conditional probabilities of the Bayesian network [27–31], having a dataset of variable observations, i.e. MCC occurrences.

#### Structural learning

In most real-world cases, such as multiple chronic condition studies, the Bayesian network structure (DAG) is partially or completely unknown and should be learned along with the conditional probabilities. Generally, the Bayesian network structure can be learned using: (1) Unsupervised learning algorithms, including score-based and constraint-based algorithms, (2) Supervised learning algorithms, where expert knowledge provides the DAG, and (3) semi-supervised methods, which combine unsupervised and supervised learning algorithms.

**Unsupervised learning algorithm** One efficient way of unsupervised structure learning algorithm begins with finding the Maximum Weight Spanning Tree (MWST) for the given data using the mutual information to provides an initial variables (node) ordering in *O*(*n*^2^) steps [32]. Next, a greedy search method such as K2 Algorithm [33] is used to incrementally form the DAG structure based on maximum likelihood in *O*(*n*^2^) steps.

**Fig 2:**
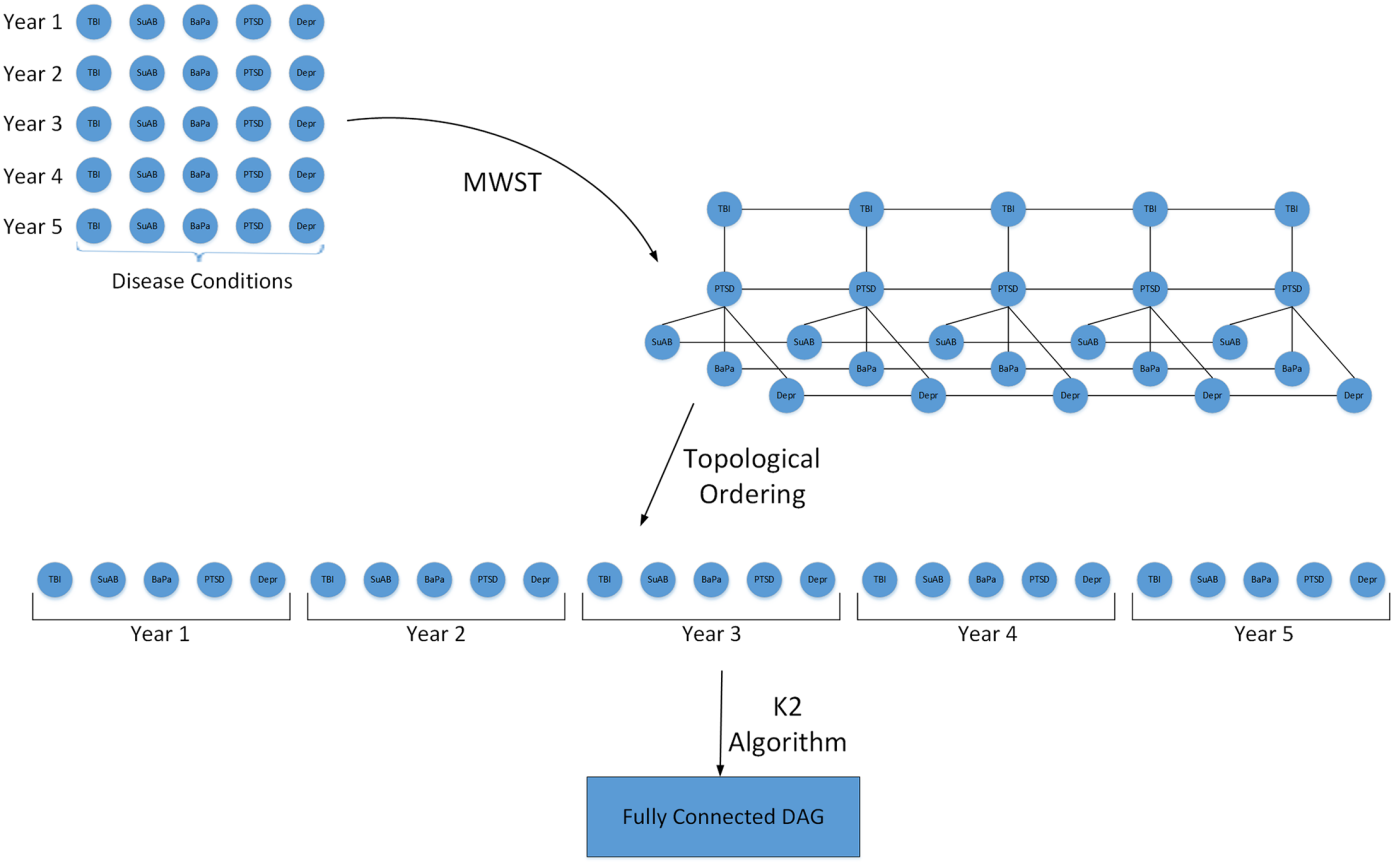
Unsupervised method. Implementation of the Proposed Algorithm (Unsupervised).

**Temporal learning** Unsupervised learning algorithm can be applied to temporal dataset as well. However, when the number of time slices and consequently variables increase, as in MCC evolution over time, the computational complexity of MWST algorithm degrades considerably. Therefore, to improve the efficiency of the learning process, we apply the MWST algorithm to each time slice separately, and then integrate the result into a single topological ordering based on their time slice (See Fig 2).

**Fig 3:**
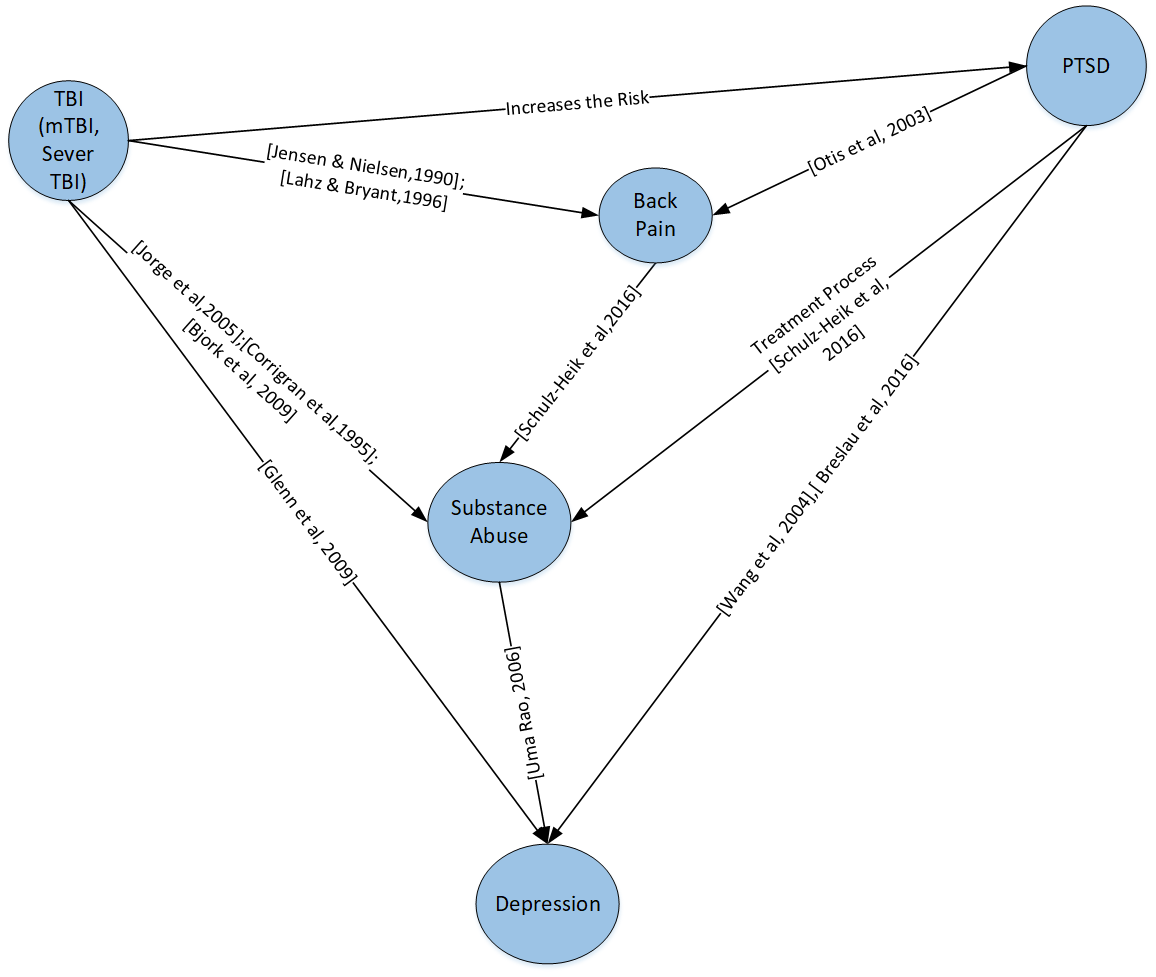
Supervised method. The network structure inferred from literature review.

**Supervised learning algorithm (expert knowledge)** It uses the expert opinion, i.e. physicians input, and/or earlier related studies to identify the complete network structure (e.g. Fig 3). Then the joint probability distributions can be calculated using either Maximum Likelihood Estimation (MLE) [27], Bayesian Estimation [28], or Expectation Maximization (EM) Algorithm (for incomplete data) [30]. In our study we use the literature review to identify the complete network structure, which is assumed to be consistent across different time slices. We also used Maximum Likelihood Parameter Estimation algorithm to calculate the conditional probabilities based in the study dataset.

**Semi-supervised method** It’s the mixture of the previous two methods. First, the initial ordering of MCC nodes are derived from the expert opinion and/or literature reviews. Next, the ordered nodes are passed through K2 algorithm to get the final structure of the network and conditional probabilities.

**Inclusion of hierarchical level** To improve the predictive performance of the Bayesian network, demographic variable such as race, sex, age group and education can be added to the DAG as higher-level variables. For this purpose, we simply connect all demographic variables to all the condition with the direction of the connections is constrained to be from demographics to chronic conditions. Meanwhile, we assume no dependence among demographic variables themselves and therefore avoiding any connection between demographics. The probability of the conditional dependencies between the demographic variables and the chronic conditions can be learned in the same we as the other conditional dependencies in the network.

#### Longest path algorithm (LPA)

Having the Bayesian network structure and probability of conditional dependencies estimated, various queries can be answered to support better decision making for practitioners to slow down or stop the progression of targeted conditions. Among the most important ones is identifying the most likely sequence of comorbidities emerging from and/or leading to specific chronic conditions, i.e. the most likely path between TBI in year 1 and substance abuse in year 5. Such query can be effectively answered by treating the preexisting condition, i.e. TBI, as the source node, and the target condition, i.e. SuAb, as the sink node, and finding the longest path between them on the graph. When only the initial chronic condition (source node), or the terminal chronic condition (sink node) is of interest, i.e. finding the most likely path (from any comorbidity) to substance abuse in year 5, we can introduce a dummy source node to the MTBN and connect it to all of the conditions in the first year, and find the longest path between the dummy source node and the sink node, i.e. SuAb in year 5, on the graph.

All the algorithms discussed in this section are included in S1 File.

## Results

### Learned bayesian structure

**Fig 4:**
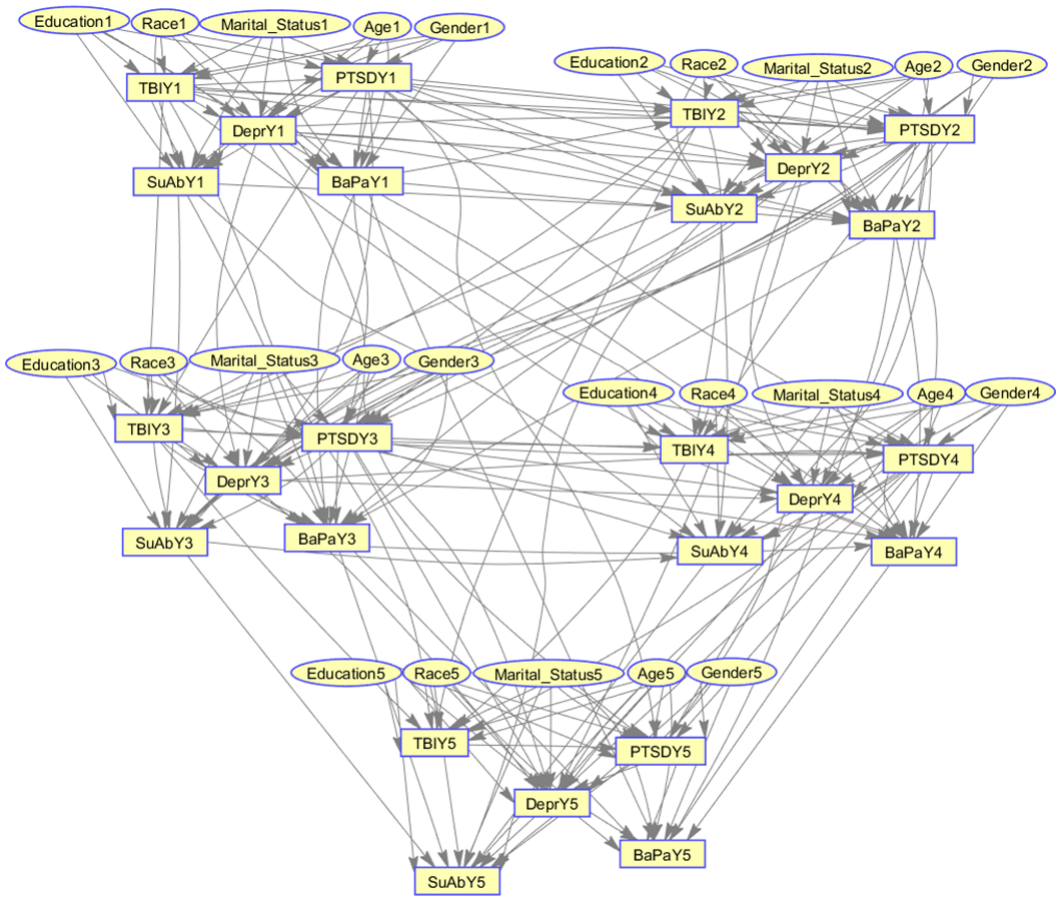
Unsupervised network. Learned BN structure from the proposed method (Unsupervised Method).

Fig 4 illustrates the proposed unsupervised Multi-level Temporal Bayesian network (MTBN). The illustrated MTBN is consisted of five-time slices, one for each year of care, five demographic variables which enable the practitioners to customize the network to specific patients, and the probabilistic dependencies among five prevalent chronic conditions in the dataset (See S1 Fig for the supervised and semi-supervised MTBNs).

Comparing the unsupervised MTBN (Fig 4) with the supervised and semi-supervised MTBN networks, we found a significant degree of similarity among the conditional dependencies as shown in Table 3.

**Table 3:** Similarity between the learned matrices.

Comparision Matrix		Supervised	semi-supervised
Unsupervised	Cosine Similarity	79.0%	93.8%
	Correlation Co-efficient	77.0%	93.0%

### Performance comparisons

We use the Area Under the Curve (AUC) of the Receiver Operating Characteristic (ROC) function [34] based on 10-fold cross validation to compare the performance of the unsupervised MTBN with the semi-supervised and supervised methods, as well as three baseline methods in the literature, including multivariate probit regression [20], multinomial logistic regression [21], and Latent Regression Markov Mixture Clustering (LRMCL) [12].

Table 4 illustrates the AUC performance of the competing methods for predicting future comorbidities, given comorbidity information of past years. For example, the first block of rows in Table 4 shows the prediction accuracy of year-2 to year 5 comorbidities, given year-1 comorbidities. Likewise, the second block of rows in Table 4 illustrates the prediction accuracy of year-3 to year 5 comorbidities, given year-1 and year-2 comorbidities. Except for the LRMCL method, which can only incorporate the comorbidity information of the immediate preceding year [12] (See first block of rows in Table 4), for all other competing methods, we collect the information of AUC performance for various years of given comorbidities, namely year 1 to year 4. As shown in Table 4, the MTBNs provides the best performance across all competing methods, followed by the probit and logistic regression and finally LRMCL. Among MTBNs, the semi-supervised learning method demonstrates the overall best performance followed by supervised learning, and unsupervised learning. Meanwhile, the unsupervised learning method which uses no expert information or supervision for structure learning, provides a comparable performance to the best performing methods. Moreover, for all of the comparing methods, as the amount of provided information (comorbidity information of past years) increases, the accuracy of the predictions increases.

**Table 4:** The Area Under the Curve (AUC) performance of the competing methods for predicting future comorbidities, given comorbidity information of past years.

	**Evidence Provided**					**Prediction Year**																			
	**Year-1**					**Year-2**					**Year-3**					**Year-4**					**Year-5**				
**DAG method**	TBI	PTSD	BaPa	SuAb	Depr	TBI	PTSD	BaPa	SuAb	Depr	TBI	PTSD	BaPa	SuAb	Depr	TBI	PTSD	BaPa	SuAb	Depr	TBI	PTSD	BaPa	SuAb	Depr
Unsupervised						72.11%	78.31%	64.28%	72.09%	66.92%	70.08%	74.95%	62.16%	69.09%	65.96%	69.19%	73.36%	55.32%	68.02%	64.70%	69.56%	71.92%	62.66%	59.41%	64.11%
Semi-Supervised						73.81%	80.10%	74.18%	79.34%	75.02%	71.20%	76.89%	70.94%	75.38%	71.21%	70.40%	75.10%	69.55%	74.60%	69.78%	70.58%	73.64%	68.21%	72.82%	68.47%
Supervised						75.08%	80.30%	74.42%	79.50%	76.42%	71.35%	76.96%	71.61%	75.58%	71.56%	70.11%	75.18%	68.61%	74.37%	68.90%	67.18%	73.61%	65.39%	72.90%	67.29%
Multinomial Logit						69.42%	72.54%	69.37%	77.16%	69.96%	66.69%	70.53%	67.03%	72.98%	64.59%	69.14%	67.75%	66.22%	72.19%	62.87%	65.46%	66.45%	64.20%	71.32%	60.97%
Multinomial Probit						69.23%	74.91%	69.81%	77.34%	70.70%	66.07%	71.78%	67.29%	73.16%	64.93%	68.50%	68.66%	66.31%	72.24%	63.10%	64.35%	67.36%	64.27%	71.44%	61.13%
LRMCL						67.59%	67.02%	66.35%	71.91%	67.34%	57.03%	63.88%	56.48%	59.54%	56.12%	51.02%	62.25%	50.18%	53.00%	49.61%	49.21%	61.18%	48.08%	42.87%	46.44%
	**Evidence Provided**										**Prediction Year**														
	**Year-1**					**Year-2**					**Year-3**					**Year-4**					**Year-5**				
**DAG method**	TBI	PTSD	BaPa	SuAb	Depr	TBI	PTSD	BaPa	SuAb	Depr	TBI	PTSD	BaPa	SuAb	Depr	TBI	PTSD	BaPa	SuAb	Depr	TBI	PTSD	BaPa	SuAb	Depr
Unsupervised											79.49%	84.29%	64.06%	75.71%	73.93%	76.17%	81.33%	55.10%	73.48%	72.35%	75.93%	79.11%	68.25%	63.92%	70.64%
Semi-Supervised											80.30%	86.37%	81.18%	85.08%	82.15%	77.03%	83.09%	77.72%	81.92%	78.47%	76.60%	80.97%	75.87%	79.33%	75.79%
Supervised											79.48%	87.11%	82.14%	85.11%	82.50%	76.77%	83.90%	77.93%	81.91%	77.68%	73.74%	81.33%	73.39%	79.65%	74.20%
Multinomial Logit											72.59%	77.03%	73.80%	81.39%	71.40%	72.28%	73.19%	71.55%	79.32%	66.83%	71.45%	71.96%	69.24%	76.24%	65.26%
Multinomial Probit											72.94%	79.79%	74.42%	81.58%	73.40%	72.12%	74.88%	71.96%	79.51%	68.45%	70.88%	73.54%	69.55%	76.46%	66.33%
	**Evidence Provided**															**Prediction Year**									
	**Year-1**					**Year-2**					**Year-3**					**Year-4**					**Year-5**				
**DAG method**	TBI	PTSD	BaPa	SuAb	Depr	TBI	PTSD	BaPa	SuAb	Depr	TBI	PTSD	BaPa	SuAb	Depr	TBI	PTSD	BaPa	SuAb	Depr	TBI	PTSD	BaPa	SuAb	Depr
Unsupervised																81.48%	85.38%	50.48%	74.11%	76.98%	79.80%	82.58%	75.06%	64.77%	74.85%
Semi-Supervised																81.52%	87.37%	82.85%	87.23%	84.39%	80.21%	84.58%	79.93%	83.50%	80.06%
Supervised																81.73%	88.60%	83.49%	87.71%	84.19%	78.71%	85.28%	79.19%	84.63%	78.99%
Multinomial Logit																76.82%	78.32%	73.52%	85.43%	71.88%	74.07%	76.57%	71.25%	79.80%	68.93%
Multinomial Probit																77.06%	80.98%	74.25%	85.76%	74.40%	73.96%	78.52%	71.77%	80.10%	70.69%
	**Evidence Provided**																				**Prediction Year**				
	**Year-1**					**Year-2**					**Year-3**					**Year-4**					**Year-5**				
**DAG method**	TBI	PTSD	BaPa	SuAb	Depr	TBI	PTSD	BaPa	SuAb	Depr	TBI	PTSD	BaPa	SuAb	Depr	TBI	PTSD	BaPa	SuAb	Depr	TBI	PTSD	BaPa	SuAb	Depr
Unsupervised																					83.47%	85.35%	81.99%	63.88%	78.35%
Semi-Supervised																					83.61%	87.10%	83.77%	87.66%	84.35%
Supervised																					82.87%	89.06%	83.83%	88.67%	84.97%
Multinomial Logit																					77.44%	79.00%	74.18%	83.22%	72.75%
Multinomial Probit																					77.66%	81.76%	74.83%	83.61%	75.31%

To demonstrate the predicted frequency of each comorbid condition, given the information of the past years, Table 5 illustrates an example of the unsupervised, semi-supervised, and supervised MTBNs’ conditional probabilities of the comorbidities in year two, given year one data, for a sample patients with the following risk factors, gender (male), marital status (unmarried), education (less than high school), race (white), and age (18-30). For the economy of space, we use the following coding system to represent the comorbidities in the table: no-comorbidity (“0”), TBI (“1”), PTSD (“2”), BaPa (“3”), SuAb (“4”), and Depr (“5”) (See S2 Table for the detail description). Also, to highlight the significance of the transition probabilities among comorbidities, higher probabilities are highlighted with darker color. To interpret the table, the numbers on the main diagonal show the probabilities of retaining the conditions, the numbers above the main diagonal present the probabilities of adding new conditions, and the numbers below the diagonal represent the probabilities of remission from one or more of the existing conditions from year one to year two. As illustrated in the table, there is a considerable similarity between the conditional probabilities and patterns of high transition probabilities of the unsupervised, semi-supervised, and supervised methods. Meanwhile, the most significant pattern of the transition probabilities across all three MTBNs is retention of the existing comorbidities, which is intuitive. The other major pattern is remission from existing conditions (numbers below the main diagonal) which is more likely to happen for small number of comorbidities, i.e. one of two.

**Table 5:** Conditional probabilities of the comorbidities in year two, given year one data, for a sample patients with the following risk factors, gender (male), marital status (unmarried), education (less than high school), race (white), and age (18-30).

**Unsupervised Method**																																
	**Year 2**																															
	**0**	**1**	**2**	**3**	**4**	**5**	**12**	**13**	**14**	**15**	**23**	**24**	**25**	**34**	**35**	**45**	**123**	**124**	**125**	**134**	**135**	**145**	**234**	**235**	**245**	**345**	**1234**	**1235**	**1345**	**1245**	**2345**	**12345**
**0**	44.2%	0.4%	15.5%	0.3%	15.1%	0.2%	3.9%	0.2%	7.6%	0.3%	1.8%	0.2%	1.8%	0.2%	0.6%	0.2%	3.1%	0.1%	0.7%	0.1%	0.7%	0.1%	0.2%	0.1%	1.2%	0.1%	0.3%	0.1%	0.3%	0.1%	0.1%	0.1%
**1**	22.0%	1.6%	18.4%	1.2%	18.2%	0.9%	9.8%	0.8%	7.5%	1.2%	2.9%	0.8%	2.9%	0.6%	1.7%	0.5%	2.3%	0.3%	0.9%	0.2%	0.9%	0.2%	0.5%	0.1%	1.4%	0.2%	0.6%	0.2%	0.6%	0.1%	0.3%	0.1%
**2**	24.5%	3.9%	12.1%	2.3%	12.1%	2.6%	4.2%	1.7%	6.2%	2.9%	2.7%	1.6%	2.8%	1.9%	1.6%	1.2%	2.8%	1.2%	1.1%	0.7%	1.2%	0.8%	0.6%	0.5%	1.6%	1.1%	0.8%	0.6%	0.9%	0.7%	0.5%	0.5%
**3**	6.8%	8.6%	9.6%	5.6%	13.0%	5.7%	6.4%	4.1%	6.1%	4.4%	3.2%	2.7%	3.5%	2.7%	2.2%	1.9%	1.9%	1.2%	1.0%	0.8%	1.1%	0.8%	0.7%	0.6%	1.2%	0.9%	0.6%	0.5%	0.7%	0.5%	0.4%	0.4%
**4**	24.7%	3.6%	13.8%	2.9%	14.4%	2.0%	5.1%	1.7%	7.5%	2.7%	3.3%	2.2%	3.0%	1.5%	1.7%	1.3%	1.3%	0.6%	0.7%	0.5%	0.5%	0.4%	0.4%	0.3%	0.8%	0.6%	0.5%	0.5%	0.4%	0.3%	0.3%	0.3%
**5**	4.5%	8.4%	12.8%	6.9%	9.0%	5.0%	6.7%	4.4%	6.2%	4.9%	4.6%	4.1%	3.4%	2.9%	2.8%	2.6%	1.1%	1.0%	0.9%	0.8%	0.6%	0.5%	0.5%	0.5%	0.9%	0.8%	0.7%	0.7%	0.5%	0.5%	0.4%	0.4%
**12**	3.6%	10.1%	8.1%	6.3%	7.9%	5.9%	4.4%	3.9%	7.0%	6.4%	4.1%	3.8%	3.9%	3.7%	2.4%	2.3%	1.7%	1.6%	1.0%	1.0%	1.0%	0.9%	0.6%	0.6%	1.4%	1.4%	0.9%	0.9%	0.8%	0.8%	0.5%	0.5%
**13**	0.5%	13.2%	9.8%	9.4%	8.7%	8.3%	6.3%	6.1%	5.0%	5.0%	3.3%	3.3%	3.1%	3.1%	2.1%	2.1%	1.2%	1.1%	0.8%	0.8%	0.7%	0.7%	0.5%	0.5%	0.9%	0.9%	0.6%	0.6%	0.5%	0.5%	0.3%	0.3%
**14**	7.8%	0.3%	10.1%	0.2%	10.5%	0.2%	3.0%	0.2%	37.1%	0.3%	7.6%	0.2%	7.9%	0.2%	2.2%	0.2%	4.0%	0.1%	0.9%	0.1%	0.9%	0.1%	0.3%	0.0%	3.6%	0.1%	0.8%	0.1%	0.8%	0.1%	0.3%	0.0%
**15**	4.8%	1.4%	12.9%	0.9%	12.7%	0.8%	7.0%	0.6%	23.2%	1.3%	8.7%	0.9%	8.4%	0.8%	4.6%	0.6%	2.2%	0.3%	0.9%	0.2%	0.9%	0.2%	0.5%	0.1%	2.0%	0.3%	0.9%	0.2%	0.8%	0.2%	0.5%	0.1%
**23**	5.9%	3.4%	9.1%	2.0%	9.7%	2.5%	3.9%	1.6%	20.6%	3.3%	6.2%	2.0%	6.7%	2.4%	2.9%	1.6%	2.9%	0.9%	1.1%	0.6%	1.2%	0.7%	0.6%	0.4%	2.6%	0.9%	1.0%	0.6%	1.1%	0.7%	0.6%	0.4%
**24**	1.8%	6.3%	6.5%	4.0%	8.7%	4.4%	4.5%	3.0%	10.9%	6.1%	5.2%	3.9%	6.3%	4.2%	3.7%	2.9%	1.9%	1.5%	1.1%	0.9%	1.2%	0.9%	0.7%	0.7%	1.8%	1.4%	1.0%	0.9%	1.1%	0.9%	0.7%	0.7%
**25**	4.9%	3.5%	9.4%	2.9%	8.7%	2.0%	3.8%	1.7%	22.0%	3.4%	7.2%	2.8%	7.1%	2.0%	3.2%	1.7%	2.3%	0.8%	1.0%	0.7%	0.8%	0.5%	0.5%	0.4%	2.2%	0.8%	1.0%	0.7%	0.8%	0.5%	0.5%	0.4%
**34**	1.6%	6.3%	9.6%	4.8%	7.0%	3.7%	4.9%	3.1%	11.7%	6.1%	7.2%	4.7%	5.7%	3.6%	4.1%	2.9%	1.4%	1.0%	1.0%	0.8%	0.7%	0.6%	0.6%	0.5%	1.3%	1.0%	0.9%	0.8%	0.7%	0.6%	0.6%	0.5%
**35**	0.8%	8.0%	6.0%	5.0%	5.7%	4.7%	3.5%	3.2%	9.5%	7.7%	5.3%	4.8%	5.1%	4.6%	3.2%	3.0%	2.0%	1.8%	1.2%	1.2%	1.1%	1.1%	0.8%	0.7%	2.0%	1.8%	1.2%	1.2%	1.1%	1.1%	0.8%	0.7%
**45**	0.3%	8.5%	6.1%	5.8%	5.7%	5.4%	3.9%	3.8%	8.5%	8.2%	5.6%	5.5%	5.2%	5.1%	3.6%	3.6%	1.4%	1.4%	1.0%	0.9%	0.9%	0.9%	0.6%	0.6%	1.4%	1.4%	0.9%	0.9%	0.9%	0.8%	0.6%	0.6%
**123**	22.7%	0.4%	12.1%	0.3%	11.9%	0.2%	3.4%	0.2%	7.5%	0.3%	1.8%	0.2%	1.8%	0.2%	0.7%	0.2%	19.3%	0.3%	4.2%	0.2%	4.1%	0.2%	1.3%	0.1%	3.7%	0.2%	0.9%	0.2%	0.9%	0.1%	0.4%	0.1%
**124**	10.2%	1.6%	13.8%	1.2%	13.4%	0.9%	7.7%	0.8%	6.6%	1.2%	2.8%	0.9%	2.7%	0.7%	1.6%	0.6%	11.3%	0.9%	4.6%	0.7%	4.3%	0.5%	2.7%	0.5%	2.7%	0.7%	1.2%	0.5%	1.2%	0.4%	0.8%	0.4%
**125**	10.8%	3.4%	8.7%	2.0%	9.1%	2.4%	3.5%	1.6%	5.3%	2.5%	2.4%	1.4%	2.5%	1.7%	1.4%	1.1%	11.7%	2.4%	3.7%	1.4%	4.0%	1.7%	1.8%	1.1%	3.2%	1.8%	1.5%	1.1%	1.6%	1.3%	1.0%	0.8%
**134**	2.5%	6.2%	6.4%	4.1%	8.5%	4.3%	4.4%	3.1%	4.8%	3.7%	2.7%	2.3%	2.9%	2.4%	1.8%	1.6%	6.3%	3.5%	3.1%	2.4%	3.8%	2.5%	2.3%	1.8%	2.6%	2.2%	1.5%	1.4%	1.6%	1.4%	1.0%	1.0%
**135**	13.3%	3.4%	10.8%	2.9%	9.9%	1.9%	4.2%	1.7%	6.4%	2.5%	3.1%	2.1%	2.6%	1.4%	1.6%	1.2%	7.7%	2.0%	3.2%	1.7%	2.1%	1.2%	1.4%	1.0%	2.6%	1.5%	1.6%	1.3%	1.2%	0.9%	0.9%	0.8%
**145**	1.6%	5.9%	7.9%	5.0%	5.5%	3.4%	4.4%	3.1%	5.0%	4.4%	4.0%	3.7%	2.8%	2.6%	2.4%	2.3%	3.9%	3.2%	3.2%	2.7%	2.3%	1.8%	1.9%	1.6%	2.6%	2.6%	2.3%	2.2%	1.5%	1.5%	1.3%	1.3%
**234**	1.5%	7.5%	5.9%	4.8%	5.5%	4.4%	3.3%	3.0%	5.0%	4.7%	3.0%	2.9%	2.9%	2.7%	1.8%	1.7%	5.1%	4.4%	3.1%	2.9%	2.8%	2.6%	1.8%	1.8%	3.0%	2.9%	1.8%	1.8%	1.7%	1.7%	1.1%	1.1%
**235**	0.2%	8.3%	6.1%	5.9%	5.3%	5.0%	3.7%	3.7%	4.6%	4.5%	3.1%	3.1%	2.8%	2.7%	1.9%	1.9%	4.3%	4.2%	3.0%	3.0%	2.5%	2.4%	1.8%	1.7%	2.7%	2.7%	1.8%	1.8%	1.6%	1.6%	1.1%	1.1%
**245**	4.0%	0.2%	7.1%	0.2%	7.5%	0.1%	2.3%	0.1%	26.6%	0.2%	5.5%	0.2%	5.6%	0.1%	1.7%	0.1%	13.4%	0.1%	2.8%	0.1%	2.9%	0.1%	0.9%	0.1%	11.7%	0.1%	2.5%	0.1%	2.5%	0.1%	0.8%	0.1%
**345**	2.4%	1.2%	9.5%	0.8%	9.3%	0.7%	5.5%	0.6%	16.9%	1.2%	6.7%	0.8%	6.5%	0.7%	3.8%	0.6%	7.1%	0.8%	3.1%	0.6%	3.0%	0.5%	1.9%	0.4%	6.2%	0.8%	2.7%	0.6%	2.6%	0.5%	1.6%	0.4%
**1234**	3.0%	2.5%	6.6%	1.6%	7.2%	1.9%	3.0%	1.2%	15.1%	2.5%	4.7%	1.5%	5.0%	1.8%	2.2%	1.2%	8.7%	1.6%	2.8%	1.0%	3.2%	1.2%	1.5%	0.8%	7.1%	1.6%	2.4%	1.0%	2.6%	1.2%	1.3%	0.8%
**1235**	0.8%	4.8%	4.6%	3.1%	5.9%	3.3%	3.2%	2.3%	7.6%	4.7%	3.9%	3.0%	4.5%	3.2%	2.8%	2.2%	4.6%	3.2%	2.5%	2.1%	2.8%	2.1%	1.8%	1.5%	4.2%	3.2%	2.4%	2.0%	2.6%	2.1%	1.7%	1.5%
**1345**	2.8%	2.7%	7.2%	2.3%	6.1%	1.6%	2.9%	1.4%	16.5%	2.7%	5.6%	2.2%	5.1%	1.6%	2.5%	1.4%	7.4%	1.6%	2.8%	1.4%	2.0%	0.9%	1.2%	0.8%	6.7%	1.6%	2.6%	1.4%	2.0%	0.9%	1.2%	0.8%
**1245**	0.8%	4.7%	7.0%	3.7%	5.0%	2.8%	3.7%	2.4%	8.3%	4.6%	5.4%	3.6%	4.1%	2.7%	3.1%	2.3%	4.0%	2.6%	2.9%	2.1%	2.1%	1.6%	1.7%	1.4%	3.7%	2.6%	2.6%	2.1%	2.0%	1.6%	1.6%	1.3%
**2345**	0.5%	5.9%	4.5%	3.8%	4.2%	3.6%	2.6%	2.4%	7.1%	5.8%	4.1%	3.8%	3.9%	3.5%	2.5%	2.4%	4.1%	3.5%	2.5%	2.3%	2.3%	2.1%	1.5%	1.5%	4.0%	3.5%	2.4%	2.3%	2.3%	2.1%	1.5%	1.5%
**12345**	0.1%	6.1%	4.4%	4.2%	4.1%	3.8%	2.8%	2.7%	6.1%	5.9%	4.2%	4.1%	3.8%	3.7%	2.7%	2.6%	3.6%	3.5%	2.5%	2.4%	2.2%	2.2%	1.6%	1.6%	3.5%	3.4%	2.4%	2.4%	2.2%	2.1%	1.5%	1.5%
**Semi-Supervised Method**																																
	**0**	**1**	**2**	**3**	**4**	**5**	**12**	**13**	**14**	**15**	**23**	**24**	**25**	**34**	**35**	**45**	**123**	**124**	**125**	**134**	**135**	**145**	**234**	**235**	**245**	**345**	**1234**	**1235**	**1345**	**1245**	**2345**	**12345**
**0**	34.1%	6.8%	4.7%	1.9%	9.1%	1.8%	1.0%	0.5%	11.5%	4.0%	2.6%	1.3%	3.3%	1.3%	0.8%	0.4%	4.1%	1.0%	0.7%	0.3%	1.5%	0.5%	0.3%	0.1%	2.6%	0.9%	0.6%	0.3%	1.1%	0.4%	0.3%	0.1%
**1**	8.0%	27.0%	4.8%	4.1%	6.5%	4.9%	1.0%	0.9%	9.4%	7.7%	2.3%	2.1%	2.7%	2.1%	0.7%	0.6%	2.8%	2.2%	0.7%	0.6%	1.0%	0.8%	0.3%	0.3%	1.8%	1.5%	0.7%	0.6%	0.8%	0.6%	0.3%	0.2%
**2**	10.3%	5.8%	25.1%	4.7%	6.4%	1.5%	4.3%	1.1%	8.1%	2.7%	6.4%	2.2%	2.3%	0.9%	1.9%	0.8%	2.9%	0.7%	2.2%	0.6%	1.1%	0.3%	0.8%	0.3%	1.8%	0.6%	1.5%	0.5%	0.8%	0.3%	0.6%	0.3%
**3**	3.7%	16.0%	16.8%	12.8%	4.1%	3.0%	2.9%	2.3%	6.0%	4.8%	4.7%	3.9%	1.7%	1.4%	1.3%	1.1%	1.8%	1.4%	1.5%	1.2%	0.7%	0.5%	0.5%	0.4%	1.2%	0.9%	0.9%	0.8%	0.5%	0.4%	0.4%	0.3%
**4**	11.0%	6.3%	3.7%	1.5%	31.3%	4.9%	2.7%	1.2%	8.8%	3.3%	2.0%	1.0%	6.8%	2.6%	1.5%	0.8%	2.2%	0.6%	0.4%	0.2%	1.9%	0.5%	0.3%	0.2%	1.3%	0.5%	0.3%	0.2%	1.2%	0.5%	0.3%	0.2%
**5**	2.2%	19.7%	3.3%	2.9%	18.8%	14.0%	2.4%	2.1%	6.4%	5.1%	1.5%	1.3%	4.9%	3.8%	1.2%	1.0%	1.3%	1.0%	0.3%	0.3%	1.1%	0.9%	0.3%	0.3%	0.8%	0.7%	0.3%	0.3%	0.7%	0.6%	0.3%	0.2%
**12**	2.9%	4.8%	17.6%	3.6%	19.1%	3.7%	12.2%	2.7%	5.4%	2.0%	4.2%	1.6%	4.2%	1.6%	3.3%	1.3%	1.3%	0.4%	1.0%	0.3%	1.2%	0.3%	0.9%	0.3%	0.8%	0.3%	0.7%	0.3%	0.7%	0.3%	0.6%	0.3%
**13**	1.0%	11.9%	11.8%	9.1%	11.9%	8.6%	8.2%	6.3%	4.0%	3.2%	3.2%	2.5%	3.1%	2.4%	2.4%	1.9%	0.8%	0.6%	0.7%	0.5%	0.7%	0.6%	0.6%	0.5%	0.5%	0.4%	0.4%	0.3%	0.5%	0.4%	0.4%	0.3%
**14**	7.5%	6.0%	4.9%	1.6%	6.4%	1.2%	0.8%	0.3%	34.8%	5.8%	4.7%	1.5%	5.7%	1.2%	0.8%	0.3%	4.8%	0.8%	0.7%	0.2%	1.4%	0.3%	0.2%	0.1%	4.6%	0.8%	0.7%	0.2%	1.3%	0.3%	0.2%	0.1%
**15**	2.4%	17.4%	3.3%	2.6%	3.9%	2.8%	0.5%	0.4%	22.2%	16.3%	3.1%	2.5%	3.6%	2.7%	0.5%	0.4%	2.9%	2.1%	0.5%	0.4%	0.8%	0.6%	0.2%	0.1%	2.8%	2.1%	0.5%	0.4%	0.8%	0.6%	0.2%	0.1%
**23**	2.8%	4.7%	16.4%	3.8%	3.6%	0.9%	2.5%	0.7%	19.9%	4.4%	14.5%	3.6%	3.2%	0.8%	2.4%	0.7%	2.8%	0.6%	2.1%	0.5%	0.8%	0.2%	0.6%	0.2%	2.6%	0.6%	2.0%	0.5%	0.8%	0.2%	0.6%	0.2%
**24**	1.2%	10.5%	10.9%	8.3%	2.4%	1.7%	1.7%	1.4%	13.4%	9.8%	10.1%	7.9%	2.2%	1.7%	1.7%	1.3%	1.7%	1.3%	1.3%	1.1%	0.5%	0.4%	0.4%	0.3%	1.7%	1.3%	1.3%	1.0%	0.5%	0.4%	0.4%	0.3%
**25**	2.4%	4.3%	2.9%	1.1%	18.0%	3.4%	2.2%	0.8%	20.6%	4.1%	2.8%	1.0%	15.4%	3.3%	2.1%	0.8%	3.0%	0.6%	0.4%	0.2%	2.4%	0.5%	0.4%	0.1%	2.8%	0.6%	0.4%	0.2%	2.3%	0.5%	0.4%	0.1%
**34**	0.8%	11.1%	2.1%	1.6%	11.4%	8.3%	1.5%	1.2%	13.8%	10.3%	1.9%	1.5%	10.3%	7.7%	1.5%	1.1%	1.8%	1.4%	0.3%	0.3%	1.5%	1.1%	0.3%	0.2%	1.8%	1.3%	0.3%	0.3%	1.5%	1.1%	0.3%	0.2%
**35**	1.0%	3.2%	10.1%	2.6%	10.7%	2.5%	7.3%	2.0%	12.3%	3.0%	9.0%	2.4%	9.2%	2.3%	6.7%	1.9%	1.8%	0.4%	1.3%	0.4%	1.5%	0.4%	1.1%	0.3%	1.7%	0.4%	1.3%	0.3%	1.4%	0.4%	1.1%	0.3%
**45**	0.4%	6.9%	6.9%	5.4%	7.0%	5.1%	5.0%	4.0%	8.5%	6.4%	6.4%	5.1%	6.3%	4.8%	4.8%	3.9%	1.1%	0.8%	0.9%	0.7%	0.9%	0.7%	0.7%	0.6%	1.1%	0.8%	0.9%	0.7%	0.9%	0.7%	0.7%	0.6%
**123**	17.3%	5.7%	4.3%	1.7%	5.5%	1.2%	0.7%	0.3%	10.5%	3.6%	2.4%	1.2%	2.2%	0.8%	0.5%	0.3%	17.9%	3.0%	2.3%	1.0%	3.1%	0.7%	0.5%	0.2%	6.1%	2.1%	1.4%	0.7%	1.5%	0.6%	0.3%	0.2%
**124**	3.0%	17.3%	3.9%	3.4%	3.3%	2.5%	0.7%	0.6%	8.7%	7.2%	2.6%	2.3%	1.8%	1.4%	0.6%	0.5%	10.0%	7.4%	2.1%	1.9%	1.9%	1.4%	0.5%	0.4%	4.7%	3.9%	1.7%	1.5%	1.1%	0.9%	0.4%	0.4%
**125**	4.9%	4.4%	18.6%	3.6%	3.6%	0.9%	2.5%	0.7%	6.9%	2.3%	5.6%	1.9%	1.4%	0.6%	1.2%	0.5%	11.6%	2.1%	8.4%	1.8%	2.0%	0.5%	1.5%	0.4%	4.0%	1.3%	3.4%	1.1%	0.9%	0.4%	0.8%	0.3%
**134**	1.4%	10.9%	11.8%	9.1%	2.2%	1.6%	1.7%	1.3%	5.7%	4.6%	4.5%	3.8%	1.2%	0.9%	0.9%	0.7%	6.5%	4.9%	5.5%	4.3%	1.2%	0.9%	1.0%	0.8%	3.1%	2.5%	2.5%	2.1%	0.7%	0.6%	0.6%	0.5%
**135**	5.6%	4.4%	2.8%	1.2%	19.8%	3.4%	2.1%	0.9%	6.9%	2.5%	1.6%	0.8%	5.4%	2.1%	1.2%	0.7%	10.5%	2.0%	1.4%	0.6%	8.5%	1.8%	1.2%	0.5%	3.6%	1.4%	0.9%	0.5%	3.3%	1.3%	0.8%	0.4%
**145**	1.0%	11.8%	2.5%	2.2%	11.1%	8.4%	1.9%	1.6%	5.7%	4.5%	1.6%	1.4%	4.4%	3.5%	1.3%	1.2%	5.8%	4.4%	1.2%	1.1%	4.8%	3.7%	1.1%	1.0%	2.8%	2.2%	1.0%	0.9%	2.5%	2.0%	0.9%	0.8%
**234**	1.5%	3.3%	11.9%	2.6%	12.3%	2.6%	8.3%	2.0%	4.3%	1.6%	3.4%	1.3%	3.4%	1.3%	2.7%	1.1%	6.5%	1.4%	4.7%	1.2%	5.3%	1.3%	3.8%	1.0%	2.2%	0.9%	1.9%	0.8%	2.1%	0.8%	1.7%	0.7%
**235**	0.5%	7.5%	7.9%	6.1%	7.3%	5.4%	5.5%	4.3%	3.7%	3.0%	3.0%	2.4%	2.9%	2.3%	2.3%	1.8%	3.8%	2.9%	3.3%	2.5%	3.2%	2.4%	2.7%	2.1%	1.8%	1.4%	1.5%	1.2%	1.7%	1.3%	1.3%	1.1%
**245**	3.7%	4.3%	3.6%	1.2%	4.5%	0.9%	0.6%	0.2%	24.5%	4.1%	3.5%	1.1%	4.1%	0.9%	0.6%	0.2%	13.4%	2.1%	1.9%	0.6%	2.6%	0.5%	0.4%	0.1%	12.2%	2.1%	1.8%	0.6%	2.5%	0.5%	0.4%	0.1%
**345**	1.1%	12.2%	2.6%	2.1%	2.8%	2.0%	0.4%	0.3%	15.8%	11.6%	2.5%	2.0%	2.6%	2.0%	0.4%	0.3%	8.0%	5.7%	1.5%	1.2%	1.6%	1.2%	0.3%	0.2%	7.6%	5.6%	1.5%	1.2%	1.6%	1.2%	0.3%	0.2%
**1234**	1.4%	3.4%	11.9%	2.8%	2.6%	0.6%	1.9%	0.5%	14.3%	3.2%	10.6%	2.7%	2.4%	0.6%	1.8%	0.5%	7.8%	1.7%	5.8%	1.4%	1.5%	0.4%	1.1%	0.3%	7.1%	1.6%	5.4%	1.4%	1.5%	0.4%	1.1%	0.3%
**1235**	0.6%	7.6%	8.0%	6.1%	1.7%	1.3%	1.3%	1.1%	9.8%	7.2%	7.6%	5.9%	1.6%	1.3%	1.3%	1.0%	5.0%	3.6%	3.8%	3.0%	1.0%	0.8%	0.8%	0.6%	4.8%	3.5%	3.8%	2.9%	1.0%	0.8%	0.8%	0.6%
**1345**	1.4%	3.1%	2.2%	0.8%	12.3%	2.4%	1.6%	0.6%	14.8%	2.9%	2.1%	0.8%	10.9%	2.3%	1.5%	0.6%	8.2%	1.6%	1.2%	0.4%	6.5%	1.3%	0.9%	0.4%	7.5%	1.5%	1.1%	0.4%	6.0%	1.3%	0.9%	0.4%
**1245**	0.5%	7.9%	1.7%	1.3%	7.9%	5.8%	1.2%	0.9%	10.0%	7.4%	1.6%	1.3%	7.3%	5.5%	1.2%	0.9%	5.2%	3.8%	0.9%	0.7%	4.1%	3.0%	0.8%	0.6%	4.9%	3.7%	0.9%	0.7%	3.9%	3.0%	0.8%	0.6%
**2345**	0.6%	2.3%	7.5%	1.9%	7.5%	1.8%	5.3%	1.4%	9.0%	2.2%	6.7%	1.8%	6.6%	1.7%	4.9%	1.4%	5.0%	1.2%	3.7%	1.0%	3.9%	1.0%	2.9%	0.8%	4.5%	1.1%	3.4%	0.9%	3.6%	0.9%	2.8%	0.8%
12345	0.2%	5.0%	5.1%	4.0%	5.0%	3.7%	3.7%	3.0%	6.3%	4.7%	4.8%	3.9%	4.6%	3.5%	3.5%	2.9%	3.3%	2.4%	2.5%	2.0%	2.6%	1.9%	2.0%	1.6%	3.1%	2.4%	2.4%	2.0%	2.5%	1.9%	2.0%	1.6%
**Supervised Method**																																
	**0**	**1**	**2**	**3**	**4**	**5**	**12**	**13**	**14**	**15**	**23**	**24**	**25**	**34**	**35**	**45**	**123**	**124**	**125**	**134**	**135**	**145**	**234**	**235**	**245**	**345**	**1234**	**1235**	**1345**	**1245**	**2345**	**12345**
**0**	33.9%	7.0%	4.6%	1.9%	9.0%	1.9%	1.0%	0.5%	11.4%	4.0%	2.6%	1.3%	3.3%	1.3%	0.7%	0.4%	4.1%	1.2%	0.7%	0.4%	1.5%	0.5%	0.3%	0.2%	2.5%	1.0%	0.6%	0.3%	1.1%	0.5%	0.3%	0.1%
**1**	7.9%	27.9%	4.9%	4.1%	6.5%	4.9%	1.0%	0.8%	9.3%	7.5%	2.3%	2.0%	2.7%	2.0%	0.7%	0.5%	2.9%	2.0%	0.8%	0.7%	1.1%	0.7%	0.3%	0.2%	1.9%	1.3%	0.7%	0.6%	0.8%	0.5%	0.3%	0.2%
**2**	9.6%	6.2%	24.0%	4.9%	6.2%	1.9%	4.2%	1.4%	8.3%	3.0%	6.6%	2.5%	2.4%	1.0%	1.9%	0.9%	2.7%	0.9%	2.1%	0.8%	1.0%	0.4%	0.7%	0.4%	1.6%	0.7%	1.3%	0.7%	0.7%	0.3%	0.5%	0.3%
**3**	3.5%	15.9%	16.4%	12.6%	4.1%	2.9%	2.9%	2.2%	6.4%	5.2%	5.0%	4.1%	1.8%	1.4%	1.3%	1.0%	1.7%	1.5%	1.5%	1.3%	0.6%	0.5%	0.4%	0.4%	1.1%	1.0%	0.9%	0.9%	0.5%	0.4%	0.3%	0.3%
**4**	11.4%	5.5%	3.8%	1.5%	32.0%	4.3%	2.8%	1.1%	9.0%	3.0%	1.9%	1.1%	6.9%	2.4%	1.4%	0.8%	2.2%	0.6%	0.4%	0.2%	1.9%	0.6%	0.3%	0.2%	1.4%	0.5%	0.3%	0.2%	1.2%	0.5%	0.3%	0.2%
**5**	3.1%	19.0%	3.3%	2.7%	19.2%	14.1%	2.3%	2.1%	6.3%	4.8%	1.4%	1.2%	4.9%	3.8%	1.1%	1.0%	1.3%	1.1%	0.3%	0.3%	1.1%	1.0%	0.3%	0.3%	0.8%	0.7%	0.3%	0.3%	0.8%	0.7%	0.3%	0.3%
**12**	3.0%	4.1%	18.5%	3.1%	19.9%	3.1%	12.8%	2.2%	6.1%	1.8%	4.8%	1.4%	4.7%	1.6%	3.7%	1.2%	1.4%	0.2%	1.0%	0.1%	1.2%	0.1%	0.9%	0.1%	0.8%	0.1%	0.6%	0.0%	0.7%	0.1%	0.6%	0.0%
**13**	1.2%	11.6%	11.8%	9.1%	11.8%	8.9%	8.3%	6.8%	4.3%	3.4%	3.2%	2.7%	3.3%	2.8%	2.4%	2.1%	0.7%	0.4%	0.5%	0.2%	0.7%	0.3%	0.5%	0.2%	0.5%	0.3%	0.3%	0.2%	0.5%	0.3%	0.3%	0.2%
**14**	7.5%	6.3%	4.7%	1.5%	6.2%	1.5%	0.8%	0.3%	33.9%	5.9%	4.5%	1.5%	5.5%	1.5%	0.8%	0.3%	4.7%	1.1%	0.7%	0.3%	1.3%	0.4%	0.2%	0.1%	4.5%	1.1%	0.6%	0.3%	1.3%	0.4%	0.2%	0.1%
**15**	2.5%	17.5%	3.3%	2.6%	3.9%	2.8%	0.6%	0.4%	22.0%	16.2%	3.1%	2.5%	3.6%	2.7%	0.5%	0.4%	2.9%	2.3%	0.5%	0.4%	0.8%	0.6%	0.2%	0.1%	2.8%	2.3%	0.5%	0.4%	0.8%	0.6%	0.2%	0.1%
**23**	2.5%	4.8%	15.9%	3.8%	3.5%	1.1%	2.5%	0.8%	19.6%	4.6%	14.3%	3.7%	3.2%	1.1%	2.4%	0.8%	2.6%	0.8%	2.0%	0.7%	0.7%	0.3%	0.6%	0.2%	2.5%	0.8%	1.9%	0.6%	0.7%	0.3%	0.6%	0.2%
**24**	1.0%	10.6%	10.7%	8.4%	2.3%	1.8%	1.8%	1.4%	13.5%	10.0%	10.1%	8.0%	2.2%	1.7%	1.7%	1.4%	1.7%	1.3%	1.3%	1.0%	0.5%	0.4%	0.4%	0.3%	1.7%	1.2%	1.3%	1.0%	0.5%	0.4%	0.4%	0.3%
**25**	2.5%	3.6%	3.0%	1.0%	18.4%	3.0%	2.3%	0.8%	21.0%	3.4%	2.9%	1.0%	15.7%	2.8%	2.2%	0.8%	3.0%	0.6%	0.5%	0.2%	2.5%	0.6%	0.4%	0.2%	2.9%	0.6%	0.5%	0.2%	2.4%	0.6%	0.4%	0.2%
**34**	1.0%	10.8%	2.1%	1.6%	11.5%	8.2%	1.6%	1.2%	13.7%	10.0%	1.9%	1.5%	10.2%	7.6%	1.5%	1.1%	1.9%	1.4%	0.4%	0.3%	1.5%	1.2%	0.3%	0.3%	1.8%	1.4%	0.4%	0.3%	1.5%	1.2%	0.3%	0.3%
**35**	0.9%	2.9%	10.3%	2.4%	10.9%	2.3%	7.5%	1.8%	12.7%	2.7%	9.3%	2.2%	9.4%	2.1%	7.0%	1.7%	1.7%	0.4%	1.3%	0.4%	1.5%	0.4%	1.1%	0.3%	1.7%	0.4%	1.3%	0.4%	1.4%	0.4%	1.1%	0.3%
**45**	0.4%	6.5%	7.0%	5.3%	6.9%	5.0%	5.2%	4.2%	8.5%	6.2%	6.5%	5.0%	6.3%	4.8%	4.9%	4.0%	1.1%	0.9%	0.9%	0.7%	0.9%	0.8%	0.7%	0.6%	1.1%	0.9%	0.9%	0.7%	0.9%	0.8%	0.7%	0.6%
**123**	16.9%	6.3%	4.1%	1.7%	5.3%	1.3%	0.7%	0.3%	10.2%	3.8%	2.4%	1.2%	2.1%	0.9%	0.5%	0.3%	17.4%	3.6%	2.2%	1.0%	3.0%	0.9%	0.5%	0.2%	6.0%	2.4%	1.4%	0.8%	1.4%	0.6%	0.3%	0.2%
**124**	2.9%	17.8%	3.9%	3.5%	3.4%	2.5%	0.7%	0.5%	9.0%	7.0%	2.6%	2.3%	1.8%	1.2%	0.6%	0.4%	10.3%	7.3%	2.1%	1.9%	1.9%	1.3%	0.5%	0.4%	4.8%	3.5%	1.7%	1.5%	1.1%	0.7%	0.4%	0.3%
**125**	4.6%	4.9%	17.3%	4.1%	3.3%	1.1%	2.3%	0.9%	6.5%	2.4%	5.4%	2.2%	1.4%	0.6%	1.1%	0.6%	10.8%	2.8%	7.8%	2.5%	1.9%	0.7%	1.4%	0.6%	3.9%	1.5%	3.4%	1.5%	0.9%	0.4%	0.7%	0.4%
**134**	1.4%	10.7%	11.2%	9.1%	2.0%	1.5%	1.4%	1.1%	5.6%	4.8%	4.6%	4.1%	1.2%	1.0%	0.8%	0.7%	6.1%	5.1%	5.4%	4.7%	1.2%	0.9%	0.8%	0.6%	3.3%	3.0%	2.8%	2.7%	0.8%	0.7%	0.5%	0.5%
**135**	5.7%	4.1%	2.8%	1.2%	19.8%	3.4%	2.1%	1.0%	6.9%	2.4%	1.5%	0.9%	5.4%	1.9%	1.2%	0.7%	10.5%	2.1%	1.4%	0.7%	8.5%	2.0%	1.2%	0.6%	3.6%	1.3%	0.9%	0.5%	3.3%	1.2%	0.8%	0.5%
**145**	1.3%	11.6%	2.3%	2.0%	11.0%	8.6%	1.7%	1.6%	5.7%	4.6%	1.6%	1.4%	4.5%	3.7%	1.3%	1.2%	5.6%	4.7%	1.0%	0.9%	4.7%	4.0%	0.9%	0.9%	2.6%	2.4%	0.9%	0.9%	2.5%	2.2%	0.9%	0.9%
**234**	1.7%	2.6%	13.4%	2.0%	13.8%	2.0%	9.4%	1.4%	4.8%	0.9%	3.8%	0.6%	3.9%	0.8%	3.0%	0.4%	7.2%	0.9%	5.3%	0.7%	5.9%	0.8%	4.4%	0.6%	2.6%	0.3%	2.1%	0.1%	2.4%	0.2%	1.9%	0.0%
**235**	0.7%	7.5%	8.6%	5.5%	8.3%	5.4%	6.1%	3.7%	4.3%	3.3%	3.0%	2.2%	3.6%	2.8%	2.4%	1.7%	4.0%	2.1%	3.1%	1.3%	3.5%	1.6%	2.7%	0.8%	2.2%	1.6%	1.3%	0.8%	2.2%	1.6%	1.3%	0.8%
**245**	3.6%	4.8%	3.4%	1.2%	4.3%	1.2%	0.6%	0.3%	23.4%	4.6%	3.2%	1.2%	3.9%	1.1%	0.6%	0.3%	12.9%	2.7%	1.7%	0.7%	2.5%	0.7%	0.4%	0.2%	11.7%	2.7%	1.7%	0.7%	2.4%	0.7%	0.4%	0.2%
**345**	1.2%	12.7%	2.5%	2.0%	2.7%	2.0%	0.5%	0.3%	15.3%	11.8%	2.4%	2.0%	2.6%	1.9%	0.5%	0.3%	7.9%	6.3%	1.4%	1.1%	1.6%	1.2%	0.3%	0.2%	7.4%	6.1%	1.4%	1.1%	1.5%	1.2%	0.3%	0.2%
**1234**	1.2%	3.8%	11.2%	3.1%	2.5%	0.8%	1.8%	0.6%	13.7%	3.7%	10.1%	3.0%	2.3%	0.8%	1.7%	0.6%	7.2%	2.2%	5.4%	1.8%	1.4%	0.5%	1.1%	0.4%	6.8%	2.1%	5.1%	1.7%	1.4%	0.5%	1.1%	0.4%
**1235**	0.5%	7.7%	7.9%	6.2%	1.7%	1.3%	1.3%	1.0%	9.9%	7.4%	7.5%	6.0%	1.6%	1.2%	1.3%	1.0%	4.9%	3.6%	3.7%	3.0%	1.0%	0.7%	0.8%	0.6%	4.8%	3.6%	3.7%	3.0%	1.0%	0.7%	0.8%	0.6%
**1345**	1.4%	2.8%	2.2%	0.8%	12.2%	2.3%	1.7%	0.7%	14.6%	2.7%	2.1%	0.8%	10.7%	2.2%	1.6%	0.6%	8.2%	1.6%	1.2%	0.5%	6.4%	1.4%	1.0%	0.4%	7.4%	1.6%	1.2%	0.5%	5.9%	1.4%	1.0%	0.4%
**1245**	0.6%	7.7%	1.7%	1.3%	7.9%	5.8%	1.3%	1.0%	9.8%	7.2%	1.6%	1.3%	7.2%	5.4%	1.2%	1.0%	5.2%	3.8%	1.0%	0.8%	4.1%	3.1%	0.8%	0.7%	4.8%	3.6%	1.0%	0.8%	3.9%	3.0%	0.8%	0.7%
**2345**	0.5%	2.2%	7.4%	1.8%	7.4%	1.7%	5.3%	1.4%	9.1%	2.1%	6.8%	1.7%	6.6%	1.6%	5.0%	1.4%	4.8%	1.2%	3.6%	0.9%	3.9%	1.0%	3.0%	0.9%	4.6%	1.2%	3.5%	0.9%	3.7%	1.0%	2.9%	0.9%
**12345**	0.2%	4.8%	5.1%	3.9%	4.8%	3.7%	3.7%	3.1%	6.2%	4.7%	4.9%	3.8%	4.6%	3.6%	3.6%	3.0%	3.1%	2.4%	2.4%	2.0%	2.5%	2.0%	2.0%	1.7%	3.1%	2.4%	2.4%	2.0%	2.4%	2.0%	2.0%	1.7%

Also to demonstrate the clinical utility of the proposed method, Table 6 provides the confusion matrices of the unsupervised, semi-supervised, and supervised MTBNs for predicting the individual comorbid conditions that may occur in year 5, given years 1 to 4 data, based on a 50% threshold. As expected from earlier results, all three structure learning methods show comparable confusion matrices, while the semi-supervised methods provides the most competitive performance. Meanwhile, the true negative rate (specificity) of all MTBNs are acceptably high, and consistent across the five comorbid conditions. But, the true positive rate (recall) of the MTBNs fluctuates in predicting new cases of TBI.

**Table 6:** The confusion matrices of the unsupervised, semi-supervised, and supervised MTBNs for prediction of comorbidities of year 5, given year 1, 2, 3, 4 data, based on a 50% threshold.

Unsupervised Method													
50% Thresholding							50% Thresholding						
TBI							PTSD						
			**Predicted Condition**							**Predicted Condition**			
		Total Population	1	0					Total Population	1	0		
		7633	Predicted Condition positive	Predicted Condition negative	Prevalence				7633	Predicted Condition positive	Predicted Condition negative	Prevalence	
			565	7068	6.54%					4368	3265	37.15%	
	1	condition positive	True Positive	False Negative	True positive rate (TPR), Sensitivity, Recall	False negative rate (FNR), Miss rate		1	condition positive	True Positive	False Negative	True positive rate (TPR), Sensitivity, Recall	False negative rate (FNR), Miss rate
**True**		499	261	238	52.30%	47.70%	**True**		2836	2600	236	91.68%	8.32%
**Condition**	0	condition negative	False Positive	True Negative	False positive rate (FPR), Fall-out	True negative rate (TNR), Specificity (SPC)	**Condition**	0	condition negative	False Positive	True Negative	False positive rate (FPR), Fall-out	True negative rate (TNR), Specificity (SPC)
		7134	304	6830	4.26%	95.74%			4797	1768	3029	36.86%	63.14%
		Accuracy	Positive predictive value (PPV), Precision	False omission rate (FOR)	Positive likelihood ratio (LR+)	Diagnostic odds ratio (DOR)			Accuracy	Positive predictive value (PPV), Precision	False omission rate (FOR)	Positive likelihood ratio (LR+)	Diagnostic odds ratio (DOR)
			46.19%	3.37%	12.2743777					59.52%	7.23%	2.487451257	
		92.90%	False discovery rate (FDR)	Negative predictive value (NPV)	Negative likelihood ratio (LR-)	24.63829611			73.75%	False discovery rate (FDR)	Negative predictive value (NPV)	Negative likelihood ratio (LR-)	18.87462612
			53.81%	96.63%	0.498182896					40.48%	92.77%	0.131788108	
50% Thresholding							50% Thresholding						
Back pain							substance abuse						
			**Predicted Condition**							**Predicted Condition**			
		Total Population	1	0					Total Population	1	0		
		7633	Predicted Condition positive	Predicted Condition negative	Prevalence				7633	Predicted Condition positive	Predicted Condition negative	Prevalence	
			4559	3074	29.83%					2730	4903	12.35%	
	1	condition positive	True Positive	False Negative	True positive rate (TPR), Sensitivity, Recall	False negative rate (FNR), Miss rate		1	condition positive	True Positive	False Negative	True positive rate (TPR), Sensitivity, Recall	False negative rate (FNR), Miss rate
**True**		2277	2052	225	90.12%	9.88%	**True**		943	506	437	53.66%	46.34%
**Condition**	0	condition negative	False Positive	True Negative	False positive rate (FPR), Fall-out	True negative rate (TNR), Specificity (SPC)	**Condition**	0	condition negative	False Positive	True Negative	False positive rate (FPR), Fall-out	True negative rate (TNR), Specificity (SPC)
		5356	2507	2849	46.81%	53.19%			6690	2224	4466	33.24%	66.76%
		Accuracy	Positive predictive value (PPV), Precision	False omission rate (FOR)	Positive likelihood ratio (LR+)	Diagnostic odds ratio (DOR)			Accuracy	Positive predictive value (PPV), Precision	False omission rate (FOR)	Positive likelihood ratio (LR+)	Diagnostic odds ratio (DOR)
			45.01%	7.32%	1.925309529					18.53%	8.91%	1.614098965	
		64.21%	False discovery rate (FDR)	Negative predictive value (NPV)	Negative likelihood ratio (LR-)	10.36413243			65.14%	False discovery rate (FDR)	Negative predictive value (NPV)	Negative likelihood ratio (LR-)	2.325160924
			54.99%	92.68%	0.185766589					81.47%	91.09%	0.694188066	
50% Thresholding													
Back pain													
			**Predicted Condition**										
		Total Population	1	0									
		7633	Predicted Condition positive	Predicted Condition negative	Prevalence								
			3213	4420	25.38%								
	1	condition positive	True Positive	False Negative	True positive rate (TPR), Sensitivity, Recall	False negative rate (FNR), Miss rate							
**True**		1937	1535	402	79.25%	20.75%							
**Condition**	0	condition negative	False Positive	True Negative	False positive rate (FPR), Fall-out	True negative rate (TNR), Specificity (SPC)							
		5696	1678	4018	29.46%	70.54%							
		Accuracy	Positive predictive value (PPV), Precision	False omission rate (FOR)	Positive likelihood ratio (LR+)	Diagnostic odds ratio (DOR)							
			47.77%	9.10%	2.690027893								
		72.75%	False discovery rate (FDR)	Negative predictive value (NPV)	Negative likelihood ratio (LR-)	9.143243852							
			52.23%	90.90%	0.294209357								
Semi-Supervised Method													
50% Thresholding							50% Thresholding						
TBI							PTSD						
			**Predicted Condition**							**Predicted Condition**			
		Total Population	1	0					Total Population	1	0		
		7633	Predicted Condition positive	Predicted Condition negative	Prevalence				7633	Predicted Condition positive	Predicted Condition negative	Prevalence	
			593	7040	6.54%					4883	2750	37.15%	
	1	condition positive	True Positive	False Negative	True positive rate (TPR), Sensitivity, Recall	False negative rate (FNR), Miss rate		1	condition positive	True Positive	False Negative	True positive rate (TPR), Sensitivity, Recall	False negative rate (FNR), Miss rate
**True**		499	273	226	54.71%	45.29%	**True**		2836	2656	180	93.65%	6.35%
**Condition**	0	condition negative	False Positive	True Negative	False positive rate (FPR), Fall-out	True negative rate (TNR), Specificity (SPC)	**Condition**	0	condition negative	False Positive	True Negative	False positive rate (FPR), Fall-out	True negative rate (TNR), Specificity (SPC)
		7134	320	6814	4.49%	95.51%			4797	2227	2570	46.42%	53.58%
		Accuracy	Positive predictive value (PPV), Precision	False omission rate (FOR)	Positive likelihood ratio (LR+)	Diagnostic odds ratio (DOR)			Accuracy	Positive predictive value (PPV), Precision	False omission rate (FOR)	Positive likelihood ratio (LR+)	Diagnostic odds ratio (DOR)
			46.04%	3.21%	12.19678106					54.39%	6.55%	2.017303981	
		92.85%	False discovery rate (FDR)	Negative predictive value (NPV)	Negative likelihood ratio (LR-)	25.72209624			68.47%	False discovery rate (FDR)	Negative predictive value (NPV)	Negative likelihood ratio (LR-)	17.02818939
			53.96%	96.79%	0.474175236					45.61%	93.45%	0.118468496	
50% Thresholding							50% Thresholding						
Back pain							substance abuse						
			**Predicted Condition**							**Predicted Condition**			
		Total Population	1	0					Total Population	1	0		
		7633	Predicted Condition positive	Predicted Condition negative	Prevalence				7633	Predicted Condition positive	Predicted Condition negative	Prevalence	
			3629	4004	29.83%					1351	6282	12.35%	
	1	condition positive	True Positive	False Negative	True positive rate (TPR), Sensitivity, Recall	False negative rate (FNR), Miss rate		1	condition positive	True Positive	False Negative	True positive rate (TPR), Sensitivity, Recall	False negative rate (FNR), Miss rate
**True**		2277	2043	234	89.72%	10.28%	**True**		943	739	204	78.37%	21.63%
**Condition**	0	condition negative	False Positive	True Negative	False positive rate (FPR), Fall-out	True negative rate (TNR), Specificity (SPC)	**Condition**	0	condition negative	False Positive	True Negative	False positive rate (FPR), Fall-out	True negative rate (TNR), Specificity (SPC)
		5356	1586	3770	29.61%	70.39%			6690	612	6078	9.15%	90.85%
		Accuracy	Positive predictive value (PPV), Precision	False omission rate (FOR)	Positive likelihood ratio (LR+)	Diagnostic odds ratio (DOR)			Accuracy	Positive predictive value (PPV), Precision	False omission rate (FOR)	Positive likelihood ratio (LR+)	Diagnostic odds ratio (DOR)
			56.30%	5.84%	3.030000648					54.70%	3.25%	8.566579336	
		76.16%	False discovery rate (FDR)	Negative predictive value (NPV)	Negative likelihood ratio (LR-)	20.75346784			89.31%	False discovery rate (FDR)	Negative predictive value (NPV)	Negative likelihood ratio (LR-)	35.97688389
			43.70%	94.16%	0.145999727					45.30%	96.75%	0.238113433	
50% Thresholding													
Back pain													
			**Predicted Condition**										
		Total Population	1	0									
		5432	Predicted Condition positive	Predicted Condition negative	Prevalence								
			3495	1937	35.66%								
	1	condition positive	True Positive	False Negative	True positive rate (TPR), Sensitivity, Recall	False negative rate (FNR), Miss rate							
**True**		1937	1698	239	87.66%	12.34%							
**Condition**	0	condition negative	False Positive	True Negative	False positive rate (FPR), Fall-out	True negative rate (TNR), Specificity (SPC)							
		3495	1797	1698	51.42%	48.58%							
		Accuracy	Positive predictive value (PPV), Precision	False omission rate (FOR)	Positive likelihood ratio (LR+)	Diagnostic odds ratio (DOR)							
			48.58%	12.34%	1.704932416								
		62.52%	False discovery rate (FDR)	Negative predictive value (NPV)	Negative likelihood ratio (LR-)	6.71319703							
			51.42%	87.66%	0.253967284								
Supervised Method													
50% Thresholding							50% Thresholding						
TBI							PTSD						
			**Predicted Condition**							**Predicted Condition**			
		Total Population	1	0					Total Population	1	0		
		7633	Predicted Condition positive	Predicted Condition negative	Prevalence				7633	Predicted Condition positive	Predicted Condition negative	Prevalence	
			341	7292	6.54%					3245	4388	37.15%	
	1	condition positive	True Positive	False Negative	True positive rate (TPR), Sensitivity, Recall	False negative rate (FNR), Miss rate		1	condition positive	True Positive	False Negative	True positive rate (TPR), Sensitivity, Recall	False negative rate (FNR), Miss rate
**True**		499	163	336	32.67%	67.33%	**True**		2836	2006	830	70.73%	29.27%
**Condition**	0	condition negative	False Positive	True Negative	False positive rate (FPR), Fall-out	True negative rate (TNR), Specificity (SPC)	**Condition**	0	condition negative	False Positive	True Negative	False positive rate (FPR), Fall-out	True negative rate (TNR), Specificity (SPC)
		7134	178	6956	2.50%	97.50%		4797	1239	3558	25.83%	74.17%
		Accuracy	Positive predictive value (PPV), Precision	False omission rate (FOR)	Positive likelihood ratio (LR+)	Diagnostic odds ratio (DOR)			Accuracy	Positive predictive value (PPV), Precision	False omission rate (FOR)	Positive likelihood ratio (LR+)	Diagnostic odds ratio (DOR)
			47.80%	4.61%	13.0918241					61.82%	18.92%	2.738565384	
		93.27%	False discovery rate (FDR)	Negative predictive value (NPV)	Negative likelihood ratio (LR-)	18.95779829			72.89%	False discovery rate (FDR)	Negative predictive value (NPV)	Negative likelihood ratio (LR-)	6.940447504
			52.20%	95.39%	0.690577244					38.18%	81.08%	0.39458052	
50% Thresholding							50% Thresholding						
Back pain							substance abuse						
			**Predicted Condition**							**Predicted Condition**			
		Total Population	1	0					Total Population	1	0		
		7633	Predicted Condition positive	Predicted Condition negative	Prevalence				7633	Predicted Condition positive	Predicted Condition negative	Prevalence	
			2794	4839	29.83%					3419	4214	12.35%	
	1	condition positive	True Positive	False Negative	True positive rate (TPR), Sensitivity, Recall	False negative rate (FNR), Miss rate		1	condition positive	True Positive	False Negative	True positive rate (TPR), Sensitivity, Recall	False negative rate (FNR), Miss rate
**True**		2277	977	1300	42.91%	57.09%	**True**		943	707	236	74.97%	25.03%
**Condition**	0	condition negative	False Positive	True Negative	False positive rate (FPR), Fall-out	True negative rate (TNR), Specificity (SPC)	**Condition**	0	condition negative	False Positive	True Negative	False positive rate (FPR), Fall-out	True negative rate (TNR), Specificity (SPC)
		5356	1817	3539	33.92%	66.08%			6690	2712	3978	40.54%	59.46%
		Accuracy	Positive predictive value (PPV), Precision	False omission rate (FOR)	Positive likelihood ratio (LR+)	Diagnostic odds ratio (DOR)			Accuracy	Positive predictive value (PPV), Precision	False omission rate (FOR)	Positive likelihood ratio (LR+)	Diagnostic odds ratio (DOR)
			34.97%	26.87%	1.264786362					20.68%	5.60%	1.849456639	
		59.16%	False discovery rate (FDR)	Negative predictive value (NPV)	Negative likelihood ratio (LR-)	1.463783498			61.38%	False discovery rate (FDR)	Negative predictive value (NPV)	Negative likelihood ratio (LR-)	4.394227164
			65.03%	73.13%	0.864052891					79.32%	94.40%	0.420883257	
50% Thresholding													
Back pain													
			**Predicted Condition**										
		Total Population	1	0									
		7633	Predicted Condition positive	Predicted Condition negative	Prevalence								
			3231	4402	25.38%								
	1	condition positive	True Positive	False Negative	True positive rate (TPR), Sensitivity, Recall	False negative rate (FNR), Miss rate							
**True**		1937	1353	584	69.85%	30.15%							
**Condition**	0	condition negative	False Positive	True Negative	False positive rate (FPR), Fall-out	True negative rate (TNR), Specificity (SPC)							
		5696	1878	3818	32.97%	67.03%							
		Accuracy	Positive predictive value (PPV), Precision	False omission rate (FOR)	Positive likelihood ratio (LR+)	Diagnostic odds ratio (DOR)							
			41.88%	13.27%	2.118568782								
		67.75%	False discovery rate (FDR)	Negative predictive value (NPV)	Negative likelihood ratio (LR-)	4.710047486							
			58.12%	86.73%	0.449797754								

### Longest path algorithm (LPA)

**Fig 5:**
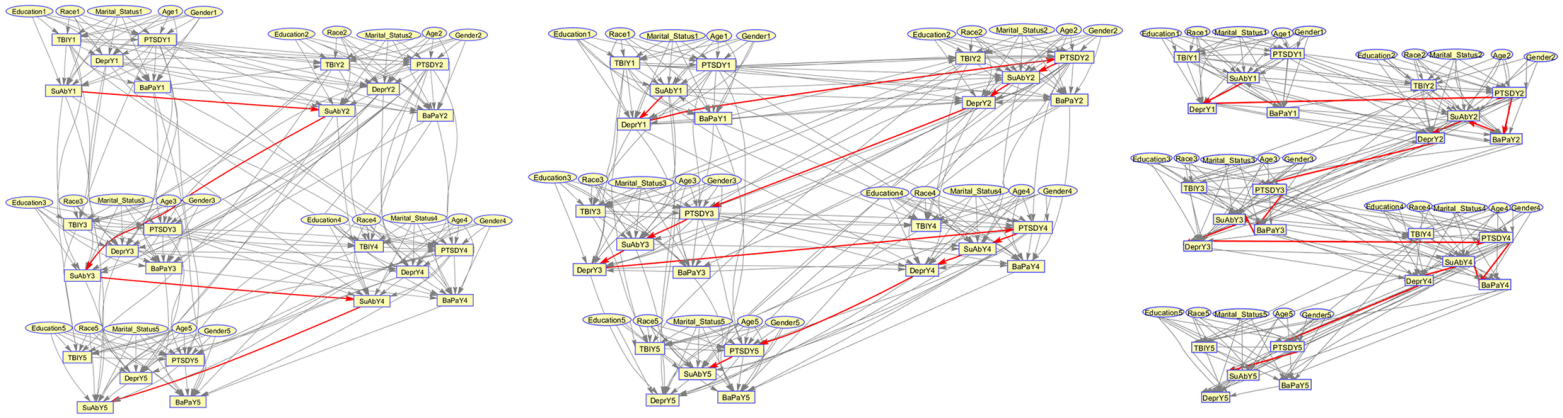
Longest path. The longest paths in the MTBNs. From left (a) Unsupervised Network (b) Semi-supervised Network and (c) Supervised Network.

Here, we use the longest path algorithm, a variation of the shortest path algorithm, to find the most likely path between the emergence of substance abuse (SuAb) in year 1, and the SUD in year 5 for the unsupervised, semi-supervised, and supervised MTBNs (Fig 5). The analysis of the longest path shows the sequence of conditions that are caused by and/or correlated with substance abuse problem at the base year and leading to diagnosis of SuAb in year 5. From the Figure, it can be seen that the most likely paths from all MTBNs, include recurrent SuAb across different years, which shows the previous history of SuAb is a major predictor of SuAb in the future. Our follow up analysis verifies the suggested sequence of SuAb in the longest path, by showing 10% of the patients with SuAb in year 5 going through it. Meanwhile, The semi-supervised and supervised Bayesian networks show additional conditions on their longest path, suggesting possible correlations between PTSD and Depr, and the continuation of substance abuse problem.

## Discussion

Clinical data are often stored as a hierarchical time series in typically large and extensive datasets. Analysis of these raw datasets may give an insight into the evolution of diseases and information about the co-occurrence of chronic conditions. This study describes an unsupervised structure learning approach for constructing Multilevel Temporal Bayesian Networks (MTBN) for mining patterns of comorbidity evolution in a large population of patients in the VA over time. Comparing the learned structure from the proposed approach with those of semi-supervised, and supervised structure learning approaches, demonstrates a significant degree of similarity between the unsupervised approach and the other learning approaches that utilize expert input. The results also show that the unsupervised approach has considerable predictive performance, which is comparable to the supervised and semi-supervised approaches, and better than multivariate probit regression, multinomial logistic regression, and Latent Regression Markov Mixture Clustering (LRMCL). Thus, in the absence of a medical expert, or sufficient literature, the unsupervised model can perform close to that of a semi or fully supervised model. The unsupervised model can also be used to generate strong hypotheses for the dependencies among chronic conditions to be tested by other approaches including clinical investigation.

We also present a Longest Path Algorithm (LPA) to mine major trajectories of comorbidities emerging from and/or leading to specific chronic conditions. We applied the LPA to mine the most probable sequence of comorbidities between the emergence of substance abuse in year 1, and substance abuse in year 5, and identified the major trajectory contains recurrent substance abuse across different years. This trajectory that includes 10% of all patients, who end up with substance abuse in year 5, suggests that the history of substance abuse is the major predictor of future substance abuse problems. Such finding from LPA might be best understood in both the context of the health care system where the diagnoses are made and the clinical associations between the diagnoses themselves. Given that substance abuse is also implicated in other adverse outcomes such as suicidality, homelessness and mortality, this use-case analysis will provide prognostic insight for clinicians caring for Veterans who sustained SuAb. In addition to that, it can be used in demonstrating utility of a method that may be used to identify and mitigate risk in individuals at greatest risk for adverse outcomes of interest such as suicide, homelessness, and early mortality.

Our findings highlight unique strengths and insights that can be gained from our algorithm and LPA and in this context, we have identified several new lines of clinical inquiry. Can early intervention of substance abuse problem prevents it in the future? Are patients with PTSD and/or Depression self-medicating with agents that predispose them to substance abuse problems, i.e., alcohol and illicit drugs? Were the treatments for these conditions suboptimal and did they include medications that predisposed patients to increased risk of substance abuse, i.e., benzodiazepines and opioids? This may be particularly relevant if back-pain is present, but not a clinical priority.

## Study limitations

In this work, patients whose data was not maintained over the focused years were omitted. Aside from death, drop out can result from not requiring care or receiving care from other health providers, which typically happens to healthier patients and those who have health insurance. Consequently, the restricted population whose incomplete data are omitted, can be biased toward healthier patients and those with health insurance, which affect the predictability of the conditions. In addition, omitting the patients with missing data and dropout considerably reduces the number of available records for estimation and validation. The validity of this study was largely dependent on the accuracy of the diagnosis and record keeping. The data used to train the model has been attained from the VA and the data set has partial bias towards conditions related to military affairs. For instance, the Veteran population with TBI is weighted toward mild TBI, and a large number with blast exposure, which is not common in the civilian population. In addition, while military service members are required to have a higher level of fitness than the general population, Veterans who receive VA care tend to be of lower socioeconomic status and have more comorbidities than Veterans who do not receive VA care. Thus, the model cannot be used for general-purpose medical analysis, and findings may not generalize beyond the study cohort. However, the model can be retrained and extended for general-purpose analysis if the model data can be extended using the public health registries with similar diagnostic factors.

## Conclusion

In this paper, we proposed an unsupervised Multi-level Temporal Bayesian Network (MTBN) for revealing the hidden patterns of interaction among multiple chronic conditions and patient levels risk factors, that can support medical decisions in absence of an medical expert or existing literature. The proposed approach develops a heuristic node ordering algorithm that reduces the computational complexity of the structure learning algorithm in temporal Bayesian networks. It also proposes a multi-level structure to model the hierarchal structure of patient level risk factors and co-morbidity. Additionally, it incorporates a Longest Path Algorithm (LPA) for identifying the most probable trajectories of comorbidities emerging from and/or leading to specific chronic conditions. We validated the performance of the proposed unsupervised approach against semi-supervised, and supervised learning Bayesian networks, as well as multivariate probit regression, multinomial logistic regression, and Latent Regression Markov Mixture Clustering (LRMCL) using a large dataset of more than 250,000 patients being monitored for 5 years. This approach has clinical implications for predicting how complex comorbid conditions may evolve. Importantly, this methodology can be used with large medical information datasets to develop predictive models for a wide variety and large number of clinical conditions including those that do not have previously demonstrated physiological or epidemiological associations.

## Supporting information

S1 Fig Semi-supervised and supervised networks. Learned BN structure from: (a) the semi-supervised method and (b) the supervised method.
(PDF)

S1 File Algorithms. The algorithms used to learn the BN structures mentioned in the manuscript.
(PDF)

S1 Table ICD-9 codes. The ICD-9 codes for the disease conditions considered in the manuscript.
(PDF)

S2 Table Disease codes in Table 5. The list of disease codes used in Table 5 of the manuscript.
(PDF)
