# Supplementary material for: Mining patterns of comorbidity evolution in patients with multiple chronic conditions using unsupervised multi-level temporal Bayesian network
Source: PLoS One. 2018 Jul 12;13(7):e0199768. doi: 10.1371/journal.pone.0199768 (PMC6042705; doi:10.1371/journal.pone.0199768)
Supplement: S1 Fig — Learned BN structure from: (a) the semi-supervised method and (b) the supervised method. (PDF) [file pone.0199768.s001.pdf]

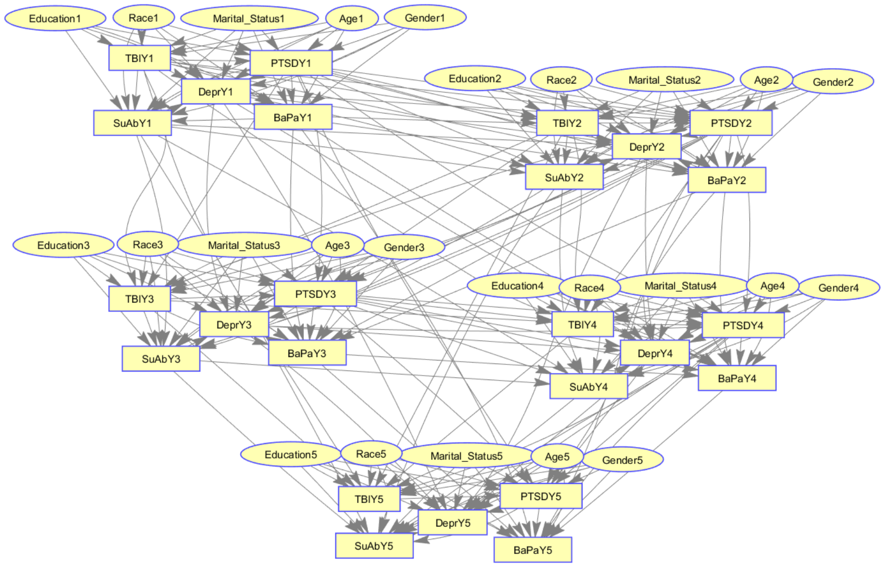

(a) Semi-supervised method.

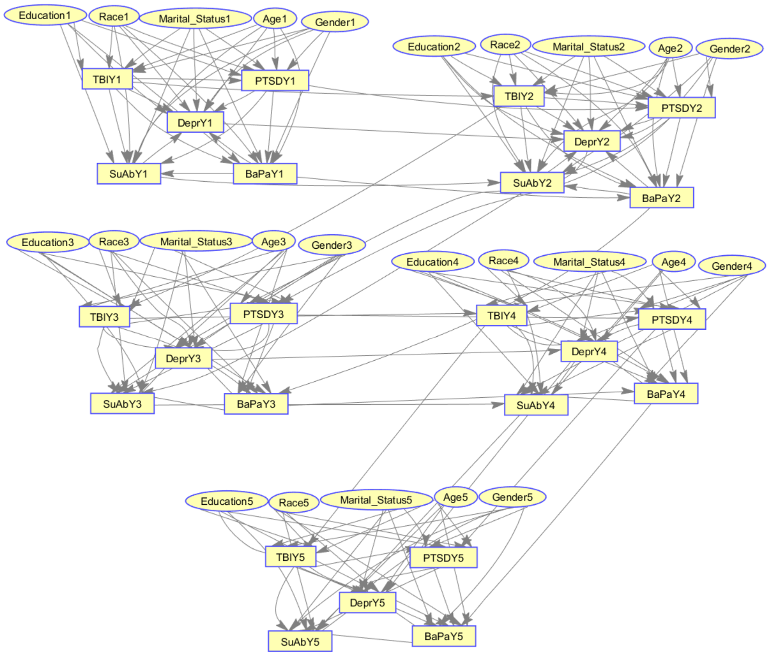

(b) Supervised method.

S1 Fig. Semi-supervised and supervised Networks. Learned BN structure from: (a) semi-supervised method and (b) supervised method.
