# Supplementary material for: Mining patterns of comorbidity evolution in patients with multiple chronic conditions using unsupervised multi-level temporal Bayesian network
Source: PLoS One. 2018 Jul 12;13(7):e0199768. doi: 10.1371/journal.pone.0199768 (PMC6042705; doi:10.1371/journal.pone.0199768)
Supplement: S1 File — The algorithms used to learn the BN structures mentioned in the manuscript. (PDF) [file pone.0199768.s002.pdf]

S1 File. Algorithms

To extend the model from a Bayesian network to Multi Level Bayesian Network the following algorithms were used. The number of patients are denoted by n. number of time slices by t_n and number of demographics by D_n. The input data has to be passed through the algorithm which possess the demographic conditions age, gender, marital status, Education and race. And the chronic conditions containing the data for TBI, Substance Abuse, PTSD, Back Pain, Depression. It is to be noted that while connecting the demographic nodes to the chronic conditions, the conditions discussed in the methodology section has to be considered. The inter relation between the chronic condition nodes has been calculated based on the K2 algorithm introduced by Cooper et al[1]. The relation between these intra and inter level nodes can be calculated using Markov Chain Monte Carlo simulations.The overall algorithm-

Algorithm MTBN: Overall Algorithm

Input: The training dataset; $(X = X_1, X_2, X_3, \dots\dots\dots, X_n)$
**1. Node Ordering:** Attain the node ordering for the condition nodes.
Option 1: Algorithm 1 & 2: Unsupervised Method
Option 2: Node ordering from Expert or Literature
**2. Algorithm 3:** Learn the Network Structure (K2 Algorithm[1])
**3. Algorithm 4:** Inclusion of Demographic Level
Output: MTBN

Maximum Weight Spanning Tree(MWST)

Algorithm 1:Maximum Weight Spanning Tree (MWST)

Input: The training dataset; $(X = X_1, X_2, X_3, \dots\dots\dots, X_n)$
**1.** for i=1 to (n-1) do
**2.** for j=2 to n do
**3.** Calculate M.I.
(Equation: $M.I.(X_i, X_j) = \sum_{i=x_i}^{n} \sum_{j=x_j}^{n} P(x_i, x_j, x_k) \log \frac{P(x_i, x_j \mid x_k)}{P(x_i) P(x_j)}$)
**4.** Pairwise Mutual Information for Weight
**5.** $E_T = MWST(I(X_i, X_j))$
**6.** end j
**7.** end i
Output: Tree Structure

MWST in Temporal Direction

Algorithm 2: MWST in Temporal Direction

Input: The training dataset; $(X = X_1, X_2, X_3, \ldots\ldots..., X_n)$

1. for k= 1 to T
2. $X_k = (D+1)\ to\ (D \times k)$
3. $E_{t_k} = MWST(X_k)$ (Algorithm 1)
4. Order= $[P(X_k)_{i,j} \in E_{t_k}]$
5. end k
6. Order Storing in one vector (Topological Sort)

Output: Node Ordering

K2 Algorithm

Algorithm 3: K2 Algorithm (As Implemented in Cooper et al[1].)

Input: The training dataset; $(X = X_1, X_2, X_3, \ldots\ldots..., X_n)$
Node ordering: Order

1. for $i = 1$ to n do
2. $\prod_i = Order(i)$
3. $\prod_i = \phi$
4. $P_old = g(i, \prod_i)$
5. OkToProceed=TRUE
6. while OkToProceed & $|\prod_i| < u$ do
7. let, z be node in Pred (Xi) - ∏i that maximizes g(i,∏i U z)
8. Pnew= g(i,∏i U z)
9. This Function is computed using equation;
$(P(B_s, D) = P(B_s) \prod_{i=1}^{n} \prod_{j=1}^{q_i} \frac{(r_i-1)!}{(N_{ij}+r_i-1)!})$
10. if $P_new > P_old$ then
11. Pold=Pnew
12. $\prod_i = \prod_i \cup \{z\}$
13. else OkToProceed=FALSE
14. end while
15. end i

Output: Node Ordering

Inclusion of Demographics Level

Algorithm 4: Inclusion of Demographic Level

Input: The training dataset and Graph; ($X = X_1, X_2, X_3, \ldots\ldots..., X_n$ & G)
**1.** for t=1 to T
**2.** for i=1 to (n-1)
**3.** for j= 2 to n
**4.** $DAG(x_i, x_j) = [D(x_t, x_j)] \cup [TBN_{DAG}(x_i, x_j)] \forall_{i,j} \in V$ (Based on conditions mentioned in the manuscript.)
**5.** end j
**6.** end i
**7.** end t
Output: Multilevel Temporal Bayesian Network (MTBN)

References

[1] Cooper, Gregory F., and Edward Herskovits. A Bayesian method for the induction of probabilistic networks from data., *Machine learning* 9.4 (1992): 309-347.
