# Supplementary material for: Mining patterns of comorbidity evolution in patients with multiple chronic conditions using unsupervised multi-level temporal Bayesian network
Source: PLoS One. 2018 Jul 12;13(7):e0199768. doi: 10.1371/journal.pone.0199768 (PMC6042705; doi:10.1371/journal.pone.0199768)
Supplement: S1 Table — The ICD-9 codes for the disease conditions considered in the manuscript. (PDF) [file pone.0199768.s003.pdf]

S1 Table. ICD-9 Codes

Table: ICD 9 Codes for the conditions selected in the manuscript.

Condition	ICD-9 Codes
TBI	800, 801, 803, 804, 850, 851, 852, 853, 854, 905.0, 907.0, 950.1, 950.2, 950.3, 959.01, 959.9, 310.2, V15.52
PTSD	309.81
Back Pain	720, 721, 722, 724, 737, 738, 739, 756, 805, 839, 847
Substance Abuse	291.xx, 292.xx, 303.x, 304.x, 305.0, 305.2, 305.3, 305.4, 305.5, 305.6, 305.7, 305.8, 305.9
Depression	296.2x, 296.3x, 311
