# Supplementary material for: Mining patterns of comorbidity evolution in patients with multiple chronic conditions using unsupervised multi-level temporal Bayesian network
Source: PLoS One. 2018 Jul 12;13(7):e0199768. doi: 10.1371/journal.pone.0199768 (PMC6042705; doi:10.1371/journal.pone.0199768)
Supplement: S2 Table — The list of disease codes used in Table 5 of the manuscript. (PDF) [file pone.0199768.s004.pdf]

S2 Table. Disease Codes in Table 5.

Table:Description of disease codes used in Table 5 of the Manuscript

Code	Description
0	No Dis
1	TBI
2	PTSD
3	Back Pain
4	Substance Abuse
5	Depression
12	TBI x PTSD
13	TBI x Back Pain
14	TBI x Substance Abuse
15	TBI x Depression
23	PTSD x Back Pain
24	PTSD x Substance Abuse
25	PTSD x Depression
34	Back Pain x Substance Abuse
35	Back Pain x Depression
45	Substance Abuse x Depression
123	TBI x PTSD x Back Pain
124	TBI x PTSD Substance Abuse
125	TBI x PTSD x Depression
134	TBI x Back Pain x Substance Abuse
135	TBI x Back Pain x Depression
145	TBI x Substance Abuse x Depression
234	PTSD x Back Pain x Substance Abuse
235	PTSD x Back Pain x Depression
245	PTSD x Substance Abuse x Depression
345	Back Pain x Substance Abuse x Depression
1234	TBI x PTSD x Back Pain x Substance Abuse
1235	TBI x PTSD x Back Pain x Depression
1345	TBI x Back Pain x Substance Abuse x Depression
1245	TBI x PTSD x Substance Abuse x Depression
2345	PTSD x Back Pain x Substance Abuse x Depression
12345	All of them
